# Testing network autocorrelation without replicates

**DOI:** 10.1371/journal.pone.0275532

**Published:** 2022-11-03

**Authors:** Kwun Chuen Gary Chan, Jinhui Han, Adrian Patrick Kennedy, Sheung Chi Phillip Yam

**Affiliations:** 1 Department of Biostatistics, University of Washington, Washington, Seattle, United States of America; 2 Department of Statistics, The Chinese University of Hong Kong, Hong Kong, China; Instituto Nacional de Medicina Genomica, MEXICO

## Abstract

In this paper, we propose a portmanteau test for whether a graph-structured network dataset without replicates exhibits autocorrelation across units connected by edges. Specifically, the well known Ljung-Box test for serial autocorrelation of time series data is generalized to the network setting using a specially derived central limit theorem for a weakly stationary random field. The asymptotic distribution of the test statistic under the null hypothesis of no autocorrelation is shown to be chi-squared, yielding a simple and easy-to-implement procedure for testing graph-structured autocorrelation, including spatial and spatial-temporal autocorrelation as special cases. Numerical simulations are carried out to demonstrate and confirm the derived asymptotic results. Convergence is found to occur quickly depending on the number of lags included in the test statistic, and a significant increase in statistical power is also observed relative to some recently proposed permutation tests. An example application is presented by fitting spatial autoregressive models to the distribution of COVID-19 cases across counties in New York state.

## Introduction

Graphical network structures are sometimes encountered by scientists in which sampling units can be regarded as vertices of a graph and units are possibly connected by edges. When independent and identically distributed (i.i.d.) replicates are observed from the graph and Gaussian assumptions are being made, the network structure can be inferred by studying the precision matrix which is the inverse of a covariance matrix. However, in many applications, replicates are unavailable and only one realization is observed on the network. A simple example is time series data in which measurements are observed through time and the measurements from two neighboring time units are connected. Typically, only a single measurement is observed at each time point and this type of data is distinguished from longitudinal data with replicates at each time point. The focus of this paper is on network data without replicates, and we avoid making explicit comparisons with methods for network data with replicates that have a vast literature. For a comprehensive discussion on graphical models with replicates, their applications and related statistical methods we refer the reader to [1, 2], while a background of multivariate dependencies in the network setting may be found in [3].

In the case of networks representing spatial relations, spatial autocorrelation might be observed in a variety of different disciplines from both the social and physical sciences. Specifically, it is common for measurements that are collected by researchers to display both serial and spatial autocorrelations; observations of a given measurement unit tend to be correlated to past measurements of that unit (serial autocorrelation) along with other closely related measurement units (spatial autocorrelation). The latter type might refer directly to the geographic proximity of two measurement units, but it could also refer to some other latent network structure. While an abundance of literature exists on testing for serial autocorrelation [4–8], comparatively few works consider the problems of testing spatial and network autocorrelation [9–11]. In this paper, we consider the problem of autocorrelation in graphical structures; that is, autocorrelation that exists in a network embedded in an undirected graph represented by vertices and edges, for which both serial and spatial autocorrelation can be viewed as a special case. It should be emphasized here that the network setting we explore is quite general; on one hand, it can represent abstract relationships such as those observed between profiles on social networking sites, and on the other hand, it can also represent a tangible spatial structure such as states or counties that directly border each other. The framework we propose should therefore find practical use in both the social and physical sciences.

Tests of no autocorrelation have been studied for some specific network structures. For time series data, Ljung and Box [12] proposed a test for serial autocorrelation which has a chi-squared asymptotic distribution under the null hypothesis of no serial autocorrelation. In the spatial statistics literature, non-parametric tests with test statistics based on Moran’s *I* coefficient are commonly used [13, 14], and this has also motivated researchers to investigate the asymptotic distribution of Moran’s *I* [15–17]. In addition, some recent works have explored non-parametric approaches to testing for spatial autocorrelation using alternative test statistics [18–21]. However, non-parametric tests generally yield smaller power than their model-based counterparts. There is also a growing recognition for the importance of accounting for network correlation, and that network and spatial data share a similar structure. For instance, Lee and Ogburn [22] used Moran’s *I* to test if the network data without replicates from the well known Framingham heart study are correlated over social network ties and whether this may lead to spurious associations if independence is assumed.

In this paper, we generalize the Ljung-Box test for serial autocorrelation to the network setting. As in the traditional Ljung-Box test, the asymptotic distribution of our test statistic is shown to be chi-squared with degrees of freedom equal to the number of lags that are included in the test statistic. The proposed network framework also immediately yields tests for spatial and spatial-temporal autocorrelation as special cases. We present comprehensive numerical simulations showing the convergence of our test statistic to the limiting chi-squared distribution, and also compare its statistical power to both the traditional non-parametric test based on Moran’s *I* and the non-parametric expected similarity and similarity entropy tests recently proposed by Farber et al. [19]. Our results demonstrate that the proposed network Ljung-Box test yields a significant improvement in terms of statistical power over that of Farber et al. [19], and a marginal improvement compared to the test based on Moran’s *I*. An example application is presented to county-level COVID-19 case figures in which we test whether the error terms arising from fitting spatial autoregressive models still exhibit network autocorrelation, which demonstrate the goodness-of-fit test of such models.

This paper is outlined as follows. First, we outline the notation and derive a central limit theorem (CLT) for estimated autocovariances associated with a network represented by an undirected graph. Second, we derive a network extension of the popular Ljung-Box test for serial autocorrelation. A numerical study of the type-I error and power of the test statistic is given to demonstrate the efficacy of the proposed test in relation to existing alternatives. Third, an application of the test in practice is presented by fitting spatial autoregressive models to COVID-19 case figures in New York counties. Finally, concluding remarks are given.

## A network central limit theorem

In this section, the notation, problem setting and a CLT for estimated network autocorrelation are presented. First, it should be emphasized that in order to parsimoniously model the network autocorrelation of a dataset, it is necessary to impose some type of structure upon it. Absent a certain network structure, a dataset consisting of *N* measurements would require the estimation of *N* × *N* covariance terms which is unachievable unless there are large numbers of replicates. The network structure we impose is represented by an undirected graph consisting of vertices and edges and the asymptotic regime we study is to let the number of vertices to grow to infinity, while having a single observation at each vertex. In the discussion that follows, the edges of the graph are assumed to be non-weighted to facilitate discussion; however, an extension to weighted graphs is readily available by suitably modifying the distance measure, which is discussed later in this paper.

Let *G* = (*V*, *E*) be an infinite undirected graph representing an appropriate super-population, in which $V={\left\{v_{k}\right\}}_{k\in Z}$ denotes the set of vertices and *E* is the set of edges. Suppose that for each vertex *v*_*i*_ ∈ *V*, there is an associated random variable *X*_*i*_ of the form

$$\begin{array}{c} X_{i}=g_{i}\left(\epsilon_{i-s},s\in Z\right),\;i\in Z \end{array}$$ (1)

where *ϵ*_*i*_, i∈Z, are a countable collection of i.i.d. random variables; $g_{i}\left(\cdot \right),i\in Z$, are measurable functions which could relate to the graph structure such as the connections between vertices. Note that all of the results presented in this paper are valid when the vertices are assumed to be located within a *d*-dimensional space and such a setting is frequently adopted in spatial statistics, but nevertheless the indices are defined to be one-dimensional for notational simplicity. Meanwhile, a special form of (1) could be *X*_*i*_ = *g*(*ϵ*_*i*_, *ϵ*_*i*−1_, ⋯), which means that in this case *X*_*i*_ does not depend on future innovations. This special form includes a variety of widely used time series models such as ARMA and GARCH processes and all the results in this paper are still valid in corresponding time series frameworks. However, we are not restricted by the network setting to a particular ordering and connection pattern between vertices. We define the distance between two vertices *v*_*i*_ and *v*_*j*_, denoted by *d*(*v*_*i*_, *v*_*j*_), as the shortest possible path between them.

To obtain a well-defined CLT in the network setting, it is necessary to define how a sequence of subgraphs $\left\{G_{n}:n\in N^{+}\right\}$ converges to the infinite graph *G*. This is particularly important since this setting is fundamentally different from the more commonly studied temporal and spatial settings equipped with an endogenous distance measure such as the Euclidean distance; in particular, the distance measure is not defined by the relative positions of the vertices, but instead by the underlying network structure. In this way, the convergence target should be known when studying the associated asymptotic statistics.

Assume that there is a sequence of subgraphs $G_{n}={\left(V_{n},E_{n}\right)}_{n\in N^{+}}\to G$ such that *V*_*n*_ ⊂ *V*, *E*_*n*_ ⊂ *E* and |*V*_*n*_| = *n*. That is, the set *V*_*n*_ is a finite subset of *V* with *n* vertices, and all the edges in the subgraph *G*_*n*_ are a part of those edges in *E*. Without loss of generality, the vertices in *V*_*n*_ are labeled as $v_{i}^{n}$, i∈Z, and the associated random variables labeled as $X_{i}^{n}$, i∈Z, with the distance $d(v_{i}^{n},v_{j}^{n})$ defined as the shortest possible path between the vertices $v_{i}^{n}$ and $v_{j}^{n}$ according to the graph *G*_*n*_ = (*V*_*n*_, *E*_*n*_). Based on the distance of two vertices, we define the following sets of *k*-distance pairs

$$\begin{array}{c} U_{k}^{n}≔\left\{\left(i,j\right):\left(v_{i}^{n},v_{j}^{n}\right)\in V_{n},\;i\leq j,\;d\left(v_{i}^{n},v_{j}^{n}\right)=k\right\}\;. \end{array}$$

The cardinality $|U_{k}^{n}|$ of the set $U_{n}^{k}$ is used to denote the number of such pairs in *G*_*n*_, with $|U_{0}^{n}|=n$. If two vertices do not share any direct or indirect connections, then their distance is infinite. The corresponding set of *k*-distance pairs is denoted as *U*_*k*_ in *G* and by construction we necessarily have $U_{k}^{n}\to U_{k}$.

In order to construct a network extension of autocorrelation measures and the Ljung-Box test statistic, it is first necessary to derive a suitable CLT for the term $S_{k}^{n}$ defined by

$$\begin{array}{c} S_{k}^{n}≔\sum_{\left(i,j\right)\in U_{k}^{n}}X_{i}X_{j}. \end{array}$$ (2)

While the terms in the sum might be dependent, the dependence structure is also different from common well-known *m*-dependence sequences [23]. To account for this dependence structure, a dependence measure is first introduced following the procedure carried out in [24]. To this end, $\left\{\epsilon_{j}^{\prime }:j\in Z\right\}$ are defined as i.i.d. copies of the random variables $\left\{\epsilon_{j}:j\in Z\right\}$, and the coupled version $X_{i}^{\left(l,*\right)}$ of *X*_*i*_ is defined as

$$\begin{array}{c} X_{i}^{\left(l,*\right)}≔g_{i}\left(\epsilon_{i-s}^{\left(l,*\right)},s\in Z\right) \end{array}$$

where

$$\begin{array}{c} \epsilon_{j}^{\left(l,*\right)}=\{\begin{array}{cc} \epsilon_{j},\; & \;\text{if}\;j\neq l\\ \epsilon_{l}^{\prime },\; & \;\text{if}\;j=l \end{array} \end{array}$$

and $X_{i}^{*}≔X_{i}^{\left(0,*\right)}$. Dependence conditions of this type are quite general and can verified in many cases [23, 25, 26].

**Definition 1 (*Physical Dependence Measure*)** *For p* ∈ (0, ∞) *and* $X_{i}\in L^{p}$, *the physical dependence measure is defined as*

$$\begin{array}{c} \delta_{l,p}≔\underset{i\in Z}{\text{sup}}{\left\|X_{i}-X_{i}^{\left(l,*\right)}\right\|}_{p},\;p\geq 1. \end{array}$$

Notice that if the function *g* = *g*_*i*_ does not depend on *i*, which means that all $X_{i},i\in Z$, have a common transformation formula of $\left\{\epsilon_{i}:i\in Z\right\}$, a simple expression can be obtained for the dependence measure as $\delta_{l,p}={\left\|X_{l}-X_{l}^{*}\right\|}_{p}$ for *p* ≥ 1. In the following, for the convenience of presentation, we adopt this simplified form so that *g* = *g*_*k*_ for all k∈Z, though the general form (1) also fits well within the following framework.

**Definition 2 (*Stability*)** *The random field* $\left\{X_{i}:i\in Z\right\}$ *of the form* (1) *is said to be p-stable if* $\Delta_{p}≔\sum_{i\in Z}\delta_{i,p}<\infty .$

As argued in [24], an input-output system can be used to interpret Eq (1) in which the i.i.d. sequence $\left\{\epsilon_{i},i\in Z\right\}$ is the input, $\left\{X_{i},i\in Z\right\}$ is the output and *g* is the underlying data generating mechanism. The data-generating structure (1) is convenient for exploring the theoretical asymptotic analysis of nonlinear stationary processes with weak dependence. The *physical dependence measure* then naturally measures the degree of nonlinear dependence of the outputs on the i.i.d. inputs, precisely, *ϵ*_0_ in the current setting and a general subset of $\left\{\epsilon_{i},i\in Z\right\}$, by applying the idea of coupling.

We further define the following sets:

$$\begin{array}{cc} \Gamma_{k}^{n} & ≔\{i:v_{i}\in V_{n}\;\text{such}\;\text{that}\;\exists v_{j}\in V_{n},i\leq j,\;\text{and}\;d\left(v_{i},v_{j}\right)=k\}\\ \Gamma_{k} & ≔\{i:v_{i}\in V\;\text{such}\;\text{that}\;\exists v_{j}\in V,i\leq j,\;\text{and}\;d\left(v_{i},v_{j}\right)=k\}\\ \Xi_{i,k}^{n} & ≔\{j:i\in \Gamma_{k}^{n}\;\text{such}\;\text{that}\;j\geq i,\;\text{and}\;d\left(v_{i},v_{j}\right)=k\}\\ \Xi_{i,k} & ≔\{j:i\in \Gamma_{k}\;\text{such}\;\text{that}\;j\geq i,\;\text{and}\;d\left(v_{i},v_{j}\right)=k\}. \end{array}$$

In the above definitions, the sets $\Gamma_{k}^{n}$ and Γ_*k*_ represent the vertices in *V*_*n*_ and *V*, respectively, that have at least one *k*-distance partner vertex. Correspondingly, the sets $\Xi_{i,k}^{n}$ and Ξ_*i*,*k*_ denote the sets of vertices that are *k*-distance apart from at least one vertex in $\Gamma_{k}^{n}$ and Γ_*k*_, respectively. We impose a weakly stationarity assumption that for any (*i*, *j*)∈*U*_*k*_, the joint moments of their associated random variables are the same up to the fourth order, and we denote *μ*_*k*_ = *E*(*X*_*i*_*X*_*j*_) for (*i*, *j*)∈*U*_*k*_. With these definitions, following the approach of [27], a suitable CLT for the term $S_{k}^{n}$ given in (2) can now be established.

**Proposition 3** *Let* $\left\{X_{i}:i\in Z\right\}$ *be a weakly stationary random field defined of the form* (1) *over the vertices V*. *Suppose that the following conditions hold*:

(i)
${\hat{\Delta }}_{k}≔\sum_{\left(i,j\right)\in U_{k}}\left(\delta_{i,4}+\delta_{j,4}\right)<\infty$ ;
(ii)
$\sigma_{n}^{2}≔E[{\left(S_{k}^{n}\right)}^{2}]\to \infty$ as *n* → ∞;
(iii)
$|\Gamma_{k}^{n}|\to \infty$ as *n* → ∞.

*Then it* *follows that*

$$\begin{array}{c} L[\frac{S_{k}^{n}-\mu_{k}}{\sqrt{|U_{k}^{n}|}},N(0,\frac{\sigma_{n}^{2}}{|U_{k}^{n}|})]⟶0 \end{array}$$ (3)

*as n* → ∞, *where* L(·,·) *is the Levy distance, and* $N(0,\sigma^{2})$ *is the normal distribution with mean 0 and variance σ*^2^.

**Corollary 4** *Assume conditions (ii) and (iii) in Proposition 3 hold*. *If* $\Xi_{i,k}^{n}=O\left(1\right)$ *and* $\sum_{i\in Z}\delta_{i,4}<\infty$, *then the conclusion of Proposition 3 holds*.

The conditions of Proposition 3 are trivially satisfied for the special case where *X*_*i*_ are i.i.d. random variables with finite fourth moment since in this case we can take *X*_*i*_ to be exactly *ϵ*_*i*_. We can also see from Corollary 4 that when each vertex in *V* has a finite number of *k*-distance partners, then the requirements in Proposition 3 are significantly simplified since in this case, we no longer need to consider the graph structure of *G* and the conditions solely relate to the dependence structure of the random field $X=\{X_{i},i\in Z\}$. With a suitable CLT derived, we are now ready to present the network extension of the traditional Ljung-Box test.

## A network Ljung-Box test

In the traditional Ljung-Box test of serial autocorrelation, the test statistic *T*(*K*) for a times series dataset of size *n* is defined in terms of the serial autocorrelations up to lag *K* as

$$\begin{array}{c} T\left(K\right)≔n\left(n+2\right)\sum_{k=1}^{K}\frac{{\hat{\rho }}_{k}^{2}}{n-k} \end{array}$$ (4)

where ${\hat{\rho }}_{k}$ is the sample autocorrelation at lag *k* = 1, 2, …, *K*. It is therefore necessary to define analogous terms which measure the network autocorrelation of a dataset that is associated with a network structure. Without loss of generality, let *X*_*i*_ be centered and have mean 0, and define the terms *r*_*n*,*k*_ by

$$\begin{array}{c} r_{k,n}≔\frac{\sum_{\left(i,j\right)\in U_{k}^{n}}X_{i}X_{j}}{\sum_{\left(i,i\right)\in U_{0}^{n}}X_{i}^{2}}. \end{array}$$ (5)

The test statistic *Q*(*K*) for the network Ljung-Box test, similar in form to *T*(*K*), is then defined as

$$\begin{array}{c} Q\left(K\right)=\sum_{k=1}^{K}\frac{n(n+\lambda -1)}{|U_{k}^{n}|}r_{k,n}^{2} \end{array}$$ (6)

where λ > 0 is a constant such that *Q*(*K*) is asymptotically chi-squared under the null hypothesis of no network autocorrelation, i.e., that *X*_*i*_ is identical to *ϵ*_*i*_ which are i.i.d. random variables. In this case, we can show that λ = *E*[(*X*_*i*_)^4^]/*E*[(*X*_*i*_)^2^]^2^ > 0, where the traditional Ljung-Box test statistic given in Eq (4) has λ = 3 as it is derived under the assumption of normally distributed random variables. Note that in the following results we assume λ is known for simplicity. In reality it would need to be estimated, however the following results will hold for any consistent estimator $\hat{\lambda }$ following suitable applications of Slutsky’s theorem to the summands in Eq (6). Further, the traditional Ljung-Box test is often used for model diagnostics by testing the absence of residual autocorrelation, where model-based residuals are used to compute the test statistics. Following the same approach as the traditional Ljung-Box test, we will show that the asymptotic distribution of *Q*(*K*) is chi-squared with *K* degrees of freedom. This is done by establishing the asymptotic normality of (5) after a suitable rescaling; indeed, the CLT of Proposition 3 is designed specifically with the numerator of (5) in mind. This gives rise to the following asymptotic result concerning the network autocorrelations.

**Theorem 5** *Let* $\left\{X_{i}:i\in Z\right\}$ *be i.i.d. centered random variables with finite fourth moment associated with the graph* *G* = (*V*, *E*). *Suppose that for k* = 1, 2, …, *K*, $|U_{k}^{n}|/n^{2}\to 0$ *as n* → ∞. *Then for k* = 1, 2, …, *K*,

$$\begin{array}{c} \sqrt{\frac{n(n+\lambda -1)}{|U_{k}^{n}|}}r_{k,n}\overset{d}{\to }N\left(0,1\right) \end{array}$$ (7)

*and*

$$\begin{array}{c} Q\left(K\right)\overset{d}{\to }\chi_{K}^{2} \end{array}$$ (8)

*as n* → ∞.

In a fully connected graph where there is an edge between every pair of vertices, $|U_{k}^{n}|=O\left(n^{2}\right)$. The requirement that $|U_{k}^{n}|=o\left(n^{2}\right)$ is mild in the sense that it still allows the number of *k*-distance pairs to grow with *n* but only at a smaller rate than a fully connected graph.

Two important yet straightforward extensions of the proposed network Ljung-Box test should be mentioned. First, if the network represents a spatial setting, then it is possible to also test for temporal autocorrelation by suitably defining the distance measure to include both time and spatial components. The second extension relates to weighted graphs; in reality, many networks are better described by weighted graphs so that the distance between two connected vertices can be taken into account. An extension for weighted graphs is readily available by use of binning, by applying a modified distance measure $\tilde{d}\left(v_{i},v_{j}\right)$ such that $\tilde{d}\left(v_{i},v_{j}\right)=k$ if *l*_*k*_ ≤ *d*(*v*_*i*_, *v*_*j*_)<*l*_*k*+ 1_, where 0 = *l*_0_ < *l*_1_ < … < *l*_*K*_, for appropriate bin values *l*_0_, *l*_1_, …, *l*_*K*_. Each vertex pair can be again described by a discrete distance which nevertheless still takes into account the weighted edges.

## Numerical study of type-I error and power

In this section, we present some simulation results to study the performance of the proposed test, and compare it to existing non-parametric tests for network autocorrelation. Since the proposed network Ljung-Box test can detect both positive and negative network autocorrelation, we consider two-tailed tests in the numerical studies.

We use the network structure from the immuno dataset from the R package igraphdata, which is an undirected network of interactions in the immunoglobulin protein, as the underlying network structure, and generate data based on that structure. In total, the immuno dataset consists of 1, 316 vertices and 6, 300 edges. We generate a sequence of increasing subgraphs from the same parent graph, i.e., the immuno dataset graph, so that the behavior observed from increasing the number of vertices is not due to changes in the underlying network structure.

**Fig 1:**
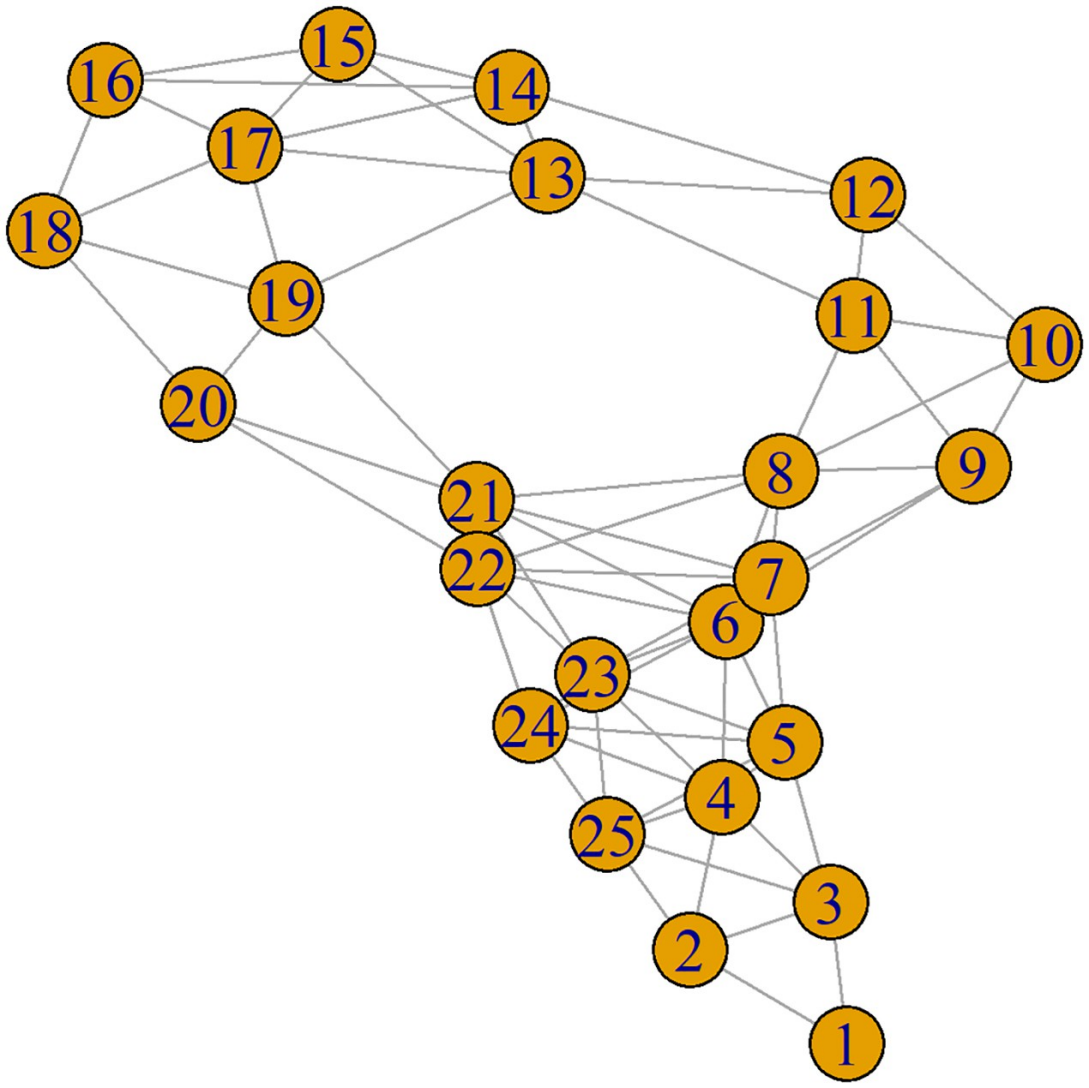
An example subgraph. The subgraph $G_{25}^{I}$ obtained from the immuno dataset using the outlined adjacency matrix procedure.

Let *G*^*I*^ denote the graph corresponding to the complete immuno dataset, and let *A*^*I*^ denote its corresponding 1316 × 1316 adjacency matrix. For a given sample size *n*, we then construct a subgraph $G_{n}^{I}$ by taking the first *n* rows and columns of the adjacency matrix *A*^*I*^; the resulting *n* × *n* submatrix $A_{n}^{I}$ is then defined as the adjacency matrix of the desired subgraph $G_{n}^{I}$. By repeating this procedure for different *n*, we obtain a sequence of subgraphs ${\left\{G_{n}^{I}\right\}}_{n}$. The subgraph $G_{25}^{I}$ that is obtained using this procedure is given in Fig 1 as an example.

For a given subgraph, to study the null distribution, the set of associated random variables *X*_*i*_ are independently generated from a standard normal N(0,1) distribution after the subgraph $G_{n}^{I}$ has been obtained. Simulations detailing convergence of the null distribution can be found in S1 Appendix, which clearly shows convergence to the asymptotic chi-squared distribution for *K* = 1, 2, 3, 4, 5, and we instead focus on the type-I error and statistical power of the test in this section. Equivalently, consideration of the statistical power will also provide insights into the type-II error rate. However, we note that convergence to the asymptotic distribution occurs faster for smaller values of *K*, as shown in S8 Fig. To provide some context on the rough number of *K*-distance interactions that these subgraphs generate, the immuno dataset with *n* = 50 yields $|U_{1}^{50}|=160$, $|U_{2}^{50}|=180$, $|U_{3}^{50}|=180$, $|U_{4}^{50}|=156$, $|U_{5}^{50}|=137$ and $|U_{6}^{50}|=124$.

We examine the statistical power and type-I error of the network Ljung-Box test, and compare the results with three existing non-parametric tests. The first test is a two-tailed test based on Moran’s *I* statistic, implemented using the moran.test function from the R package spdep, which calculates the Moran’s *I* statistic for a given weight matrix *W* and returns a *p*-value based on an estimated *Z*-score. More information on the test can be found in the online spdep package documentation. We consider three different weight matrices: (i) *W*_1_ is the adjacency matrix for the network; (ii) *W*_2_ has entries $w_{ij}^{\left(2\right)}=1,0.5$ if vertices *v*_*i*_, *v*_*j*_ are distances *d*(*v*_*i*_, *v*_*j*_) = 1, 2 apart, respectively, and $w_{ij}^{\left(2\right)}=0$ otherwise; and (iii) *W*_3_ has entries $w_{ij}^{\left(3\right)}=1,0.5,0.25$ if vertices *v*_*i*_, *v*_*j*_ are distances *d*(*v*_*i*_, *v*_*j*_) = 1, 2, 3 apart, respectively, and $w_{ij}^{\left(3\right)}=0$ otherwise. The remaining two non-parametric tests considered are those based on expected similarity and similarity entropy [19].

To numerically estimate the power and type-I error of each test, we generate random variables *ϵ*_*j*_ independently from a standard normal distribution, for *j* = 1, 2, …, *n*, and consider the transformed random variables

$$\begin{array}{c} X_{j}=\epsilon_{j}+b\frac{\sum_{k\in \Xi_{j}}\epsilon_{j}}{|\Xi_{j}^{n}|} \end{array}$$ (9)

as our associated random variables, where $\Xi_{j}^{n}$ is the set of vertices in the immuno subgraph $G_{n}^{I}$ that are neighbors of vertex *j*. Clearly, *b* = 0 corresponds to network independence, and increasing (resp. decreasing) *b* leads to stronger positive (resp. negative) network autocorrelation between the random variables defined by (9). For each *n* and *b* considered, we generate 5, 000 replications of the above sets of random variables and test for network autocorrelation using network lags *K* = 1, 2, 3, 4 for the network Ljung-Box test that we propose, weight matrices *W*_1_, *W*_2_ and *W*_3_ for the Moran’s *I* test, along with the expected similarity and similarity entropy tests proposed by Farber et al. [19]. The tests are conducted at the 5% level of significance, i.e., network independence is rejected if the resulting *p*-value is less than 0.05. The statistical power and type-I error of each test is then estimated as the proportion of those 5, 000 generated samples that reject the null.

Although the expected similarity and similarity entropy tests are primarily designed for discrete or categorical variables, Farber et al. [19] proposed it be extended to continuous variables by binning according to certain quantiles. We therefore implement these non-parametric tests by assigning labels to the random variables *X*_*i*_ according to which of the following bins they belong to: [−∞, −0.67), [−0.67, 0), [0, 0.67) and [0.67, ∞). Note that these bins were chosen so as to correspond to the quantiles of the standard normal distribution. A similarity relation is then defined on the network by saying two neighboring vertices are similar if their associated random variables lie within the same bin, while the test statistics are the sample expected similarity and the sample similarity entropy; readers are referred to [19] for details on these non-parametric tests, test statistics, and the background discussion. Within each of the 5, 000 generated samples, the two-tailed *p*-values for the permutation tests are obtained using 1,000 permutation resamples.

To examine the type-I error, we set *b* = 0 so that there is no network autocorrelation in the generated sets of associated random variables. The resulting type-I error rates for all tests are summarized in Table 1 and shown in S1 Fig of the online supplementary material. For the network Ljung-Box test, we find that the type-I error is close to 0.05 for *K* = 1, but increases non-negligibly with the number of network lags *K*. However, as *n* increases, the type-I error approaches 0.05 for even the higher order lags. Importantly, even for small *n*, the type-I error is still reasonably close to the nominal value. As expected, the type-I error of the non-parametric tests are also close to 0.05 for all *K* and *n*.

**Table 1:** Type-I error rates. Type-I error rates of tests for different values of *K* and *n*.

Type-I Error (*b* = 0)									
	Moran’s *I*			Farber et al. (2015)		Network Ljung-Box Test			
	*W*_1_	*W*_2_	*W*_3_	Expected Similarity	Similarity Entropy	*K* = 1	*K* = 2	*K* = 3	*K* = 4
*n* = 25	0.051	0.049	0.047	0.045	0.050	0.049	0.059	0.069	0.074
*n* = 50	0.043	0.041	0.040	0.051	0.050	0.049	0.053	0.062	0.068
*n* = 75	0.050	0.049	0.049	0.047	0.049	0.050	0.054	0.061	0.070
*n* = 100	0.044	0.044	0.045	0.051	0.056	0.047	0.053	0.061	0.068
*n* = 125	0.051	0.047	0.048	0.050	0.058	0.048	0.055	0.060	0.066
*n* = 150	0.044	0.044	0.040	0.049	0.047	0.049	0.050	0.060	0.067
*n* = 175	0.044	0.043	0.039	0.049	0.049	0.043	0.054	0.058	0.063
*n* = 200	0.050	0.048	0.048	0.047	0.046	0.054	0.058	0.057	0.063
*n* = 225	0.046	0.051	0.047	0.054	0.051	0.051	0.053	0.056	0.063
*n* = 250	0.050	0.044	0.041	0.046	0.051	0.049	0.050	0.053	0.060

We next investigate how the statistical power varies with both the number of vertices *n* and the parameter *b* that controls the strength and sign of the network autocorrelation. First, we fix *b* = 0.5 to investigate the case of positive network autocorrelation and calculate the statistical power of each test for different values of *n*, as summarized in Table 2 and shown in S2 Fig of the supplementary material. The statistical power of the network Ljung-Box test, for all lags and for all values of *n*, is found to be significantly higher than both the expected similarity and similarity entropy tests. Specifically, the powers of the network Ljung-Box test for the network lags *K* = 1, 2, 3, 4 are between 0.330 and 0.344 for *n* = 25, compared to just 0.137 and 0.091 for the expected similarity and similarity entropy tests, respectively. This gap in power is maintained, and indeed widens slightly, as we increase the number of vertices *n*. The network Ljung-Box test with lag *K* = 1 also has higher power than the Moran’s *I* tests for all values of *n*, though the difference is marginal when the weight matrix *W*_1_ is used; given the large discrepancy in power between *W*_1_ and *W*_2_, *W*_3_, the performance of Moran’s *I* test appears more dependent on the choice of weight matrix, whereas the network Ljung-Box test maintains high power for multiple lags *K*. For small sizes of *n* there does not appear to be any marked difference in power across the values of *K* considered; however, as *n* increases, we observe that smaller network lags *K* begin to demonstrate relatively higher power, with *K* = 1 and *K* = 4 yielding powers of 0.926 and 0.868 when *n* = 250, respectively. This discrepancy might be due to the fact that the correlated random variables *X*_*i*_ defined by (9) depend directly only on their immediate neighbors, and only indirectly on their *K*-distance neighbors for *K* > 1, so that increasing the number of network lags does not meaningfully increase the information included in the test statistic.

**Table 2:** Statistical power for positive network autocorrelation. Statistical power of tests for different values of *n* and *K* with *b* = 0.5.

Statistical Power (*b* = 0.5)									
	Moran’s *I*			Farber et al. (2015)		Network Ljung-Box Test			
	*W*_1_	*W*_2_	*W*_3_	Expected Similarity	Similarity Entropy	*K* = 1	*K* = 2	*K* = 3	*K* = 4
*n* = 25	0.308	0.284	0.275	0.137	0.091	0.344	0.330	0.337	0.352
*n* = 50	0.464	0.394	0.394	0.204	0.129	0.508	0.471	0.469	0.472
*n* = 75	0.517	0.429	0.423	0.215	0.131	0.546	0.515	0.503	0.504
*n* = 100	0.579	0.449	0.437	0.233	0.151	0.615	0.578	0.558	0.555
*n* = 125	0.684	0.526	0.516	0.283	0.162	0.696	0.656	0.625	0.614
*n* = 150	0.745	0.598	0.565	0.329	0.176	0.775	0.725	0.696	0.688
*n* = 175	0.819	0.643	0.603	0.381	0.207	0.834	0.798	0.775	0.757
*n* = 200	0.835	0.672	0.628	0.403	0.230	0.853	0.816	0.793	0.781
*n* = 225	0.889	0.727	0.677	0.451	0.242	0.898	0.864	0.845	0.831
*n* = 250	0.917	0.769	0.723	0.502	0.272	0.926	0.899	0.884	0.868

Equivalently, owing to the inverse relationship between power and type-II error, the results in Table 2 show that the network Ljung-Box test with *K* = 1 has the lowest type-II error rate among all of the tests considered. The reduction in type-II error is significant when compared to the tests proposed in [19], but only marginal compared to Moran’s *I* with weight matrix *W*_1_.

We repeat the above simulation with *b* = −0.5 to separately investigate the case of negative network autocorrelation. The results are summarized in Table 3 and shown in S3 Fig of the supplementary material. We again find a significant increase in power compared to the expected similarity and similarity entropy tests. For small *n*, this improvement in power is evident only for small network lags. However, this improvement in power increases significantly as the numbers of vertices *n* is increased; for *n* = 250 the expected similarity and similarity entropy tests have powers of just 0.346 and 0.163, respectively, while the network Ljung-Box test with powers *K* = 1 and *K* = 4 have powers 0.974 and 0.719, respectively. While the network Ljung-Box test with lag *K* = 1 again has higher power than the Moran’s *I* test for all values of *n*, once again the improvement is marginal when using the weight matrix *W*_1_. As a result, we conclude that while the proposed network Ljung-Box test does have higher power than the widely used Moran’s *I* test for both positive and negative network autocorrelation, the improvement appears marginal. On the other hand, the discrepancy in power between *W*_1_ and *W*_2_, *W*_3_ again suggests that the Moran’s *I* test is sensitive to the choice of weight matrix, whereas the network Ljung-Box test is comparatively more robust. For large *n*, larger network lags *K* also yield a significant decrease in power. As in the preceding example with *b* = 0.5, we note that the reduction in power as *K* increases might be caused by the fact that the transformed variables *X*_*i*_ depend directly only on their immediate neighbors.

**Table 3:** Statistical power for negative network autocorrelation. Statistical power of tests for different values of *n* and *K* with *b* = −0.5.

Statistical Power (*b* = −0.5)									
	Moran’s *I*			Farber et al. (2015)		Network Ljung-Box Test			
	*W*_1_	*W*_2_	*W*_3_	Expected Similarity	Similarity Entropy	*K* = 1	*K* = 2	*K* = 3	*K* = 4
*n* = 25	0.097	0.004	0.002	0.066	0.055	0.125	0.067	0.055	0.051
*n* = 50	0.259	0.044	0.036	0.114	0.072	0.316	0.149	0.091	0.069
*n* = 75	0.371	0.089	0.088	0.130	0.071	0.414	0.197	0.117	0.081
*n* = 100	0.502	0.126	0.122	0.145	0.081	0.522	0.286	0.179	0.114
*n* = 125	0.644	0.290	0.286	0.194	0.089	0.700	0.446	0.303	0.206
*n* = 150	0.764	0.435	0.418	0.225	0.111	0.801	0.566	0.405	0.287
*n* = 175	0.863	0.565	0.512	0.249	0.120	0.872	0.681	0.518	0.393
*n* = 200	0.890	0.649	0.575	0.271	0.124	0.907	0.768	0.620	0.487
*n* = 225	0.932	0.749	0.715	0.310	0.146	0.944	0.848	0.723	0.603
*n* = 250	0.966	0.848	0.828	0.346	0.163	0.974	0.917	0.824	0.719

Similarly, Table 3 shows that when the network autocorrelation is negative, the network Ljung-Box test with *K* = 1 again has the lowest type-II error rate. However, while there is a substantial reduction compared to the expected similarity and similarity entropy tests, the reduction is only marginal when compared to the Moran’s *I* test with weight matrix *W*_1_.

**Fig 2:**
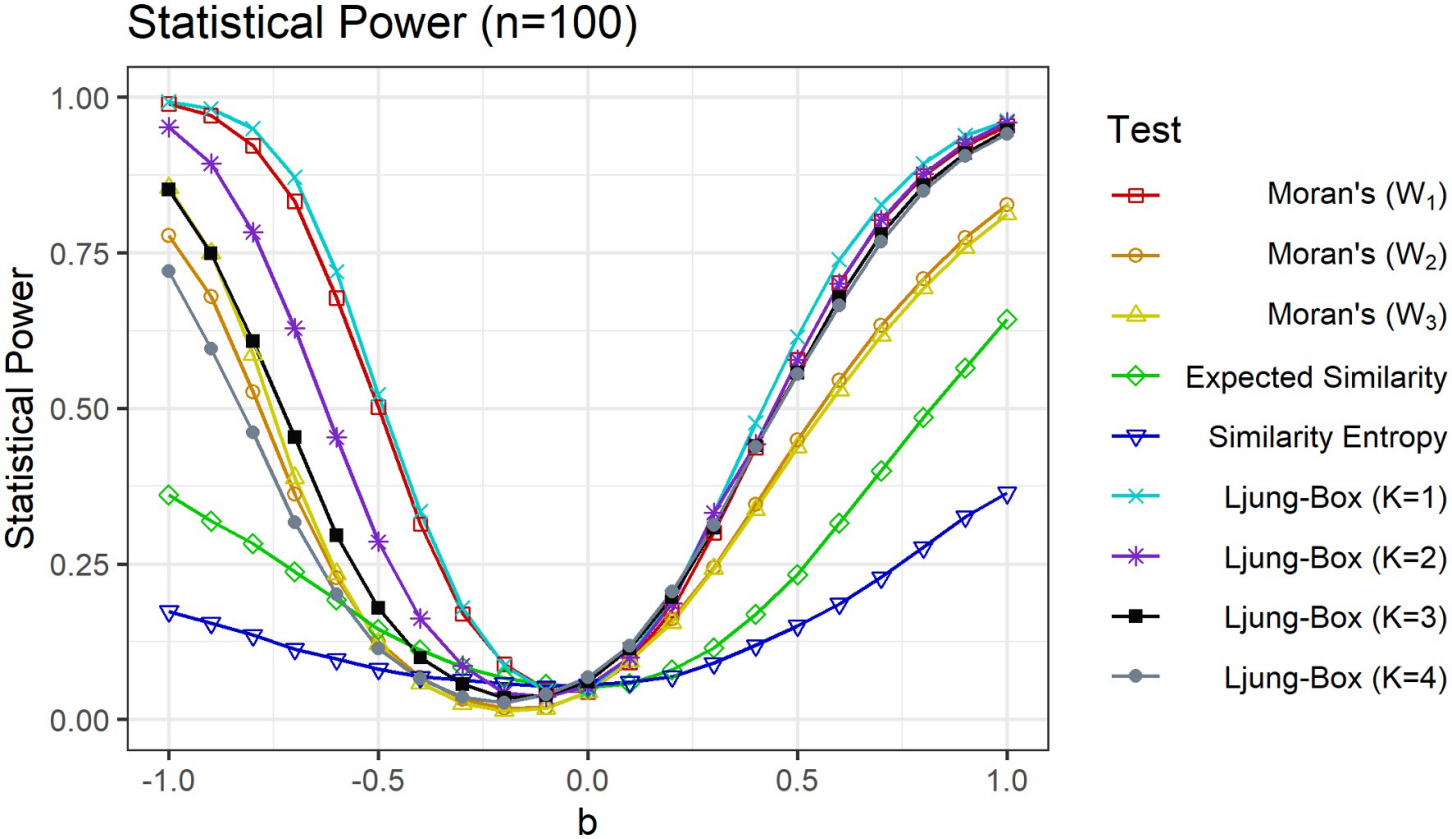
Statistical power vs. network autocorrelation. Statistical power of the network Ljung-Box test for network lags *K* = 1, 2, 3, 4 compared to expected similarity, similarity entropy and Moran’s *I* tests for *n* = 100 and −1.0 ≤ *b* ≤ 1.0.

Next, to further understand how the statistical power changes with strength and sign of the network autocorrelation, we fix *n* = 100 and vary the parameter *b* from −1.0 to 1.0. For each value of *b*, the statistical power is estimated using the same simulation approach as outlined above. The results are shown in Fig 2 and are summarized in Table 4. A striking asymmetry between positive and negatives network autocorrelation is immediately evident. As we increase *b* from 0 to 1.0, the power of the network Ljung-Box test increases significantly for small network lags *K*, with all lags yielding significantly larger power than the expected similarity and similarity entropy permutation tests. In contrast, as we decrease *b* from 0 to −1.0, the statistical power again increases for all network lags *K*, but the difference widens markedly such that smaller lags *K* yield noticeably higher statistical power. This asymmetry might be due in part to the fact that under negative autocorrelation the network assumes a checkerboard pattern with highly dissimilar values; the transitive nature of correlation would then lead even and odd lags to tend to have opposite signs, such that including multiple lags effectively cancels each other out in the test statistic. These results suggest that the choice of the number of network lags *K* to include in the test statistic *Q*(*K*) might depend crucially on whether negative or positive network autocorrelation is suspected; specifically, if negative autocorrelation is suspected, then *K* should be kept small. Finally, we again find that the network Ljung-Box test with lag *K* = 1 has marginally higher power than the Moran’s *I* test with weight matrix *W*_1_ for most values of *b*.

**Table 4:** Statistical power vs. network autocorrelation. Statistical power of tests for different values of *b* with *n* = 100.

Statistical Power (*n* = 100)									
	Moran’s *I*			Farber et al. (2015)		Network Ljung-Box Test			
	*W*_1_	*W*_2_	*W*_3_	Expected Similarity	Similarity Entropy	*K* = 1	*K* = 2	*K* = 3	*K* = 4
*b* = −1.0	0.990	0.778	0.854	0.361	0.174	0.992	0.952	0.851	0.721
*b* = −0.9	0.970	0.680	0.750	0.319	0.155	0.982	0.894	0.749	0.595
*b* = −0.8	0.922	0.526	0.587	0.283	0.136	0.950	0.783	0.608	0.461
*b* = −0.7	0.833	0.362	0.389	0.238	0.113	0.871	0.629	0.454	0.317
*b* = −0.6	0.677	0.228	0.234	0.192	0.097	0.720	0.453	0.296	0.202
*b* = −0.5	0.502	0.126	0.122	0.145	0.081	0.522	0.286	0.179	0.114
*b* = −0.4	0.314	0.068	0.059	0.112	0.069	0.335	0.163	0.099	0.066
*b* = −0.3	0.170	0.033	0.026	0.085	0.063	0.179	0.086	0.056	0.036
*b* = −0.2	0.089	0.018	0.014	0.067	0.058	0.086	0.042	0.035	0.027
*b* = −0.1	0.048	0.020	0.018	0.057	0.053	0.046	0.037	0.039	0.040
*b* = 0	0.044	0.044	0.045	0.051	0.056	0.047	0.053	0.061	0.068
*b* = 0.1	0.092	0.093	0.094	0.058	0.060	0.100	0.100	0.114	0.118
*b* = 0.2	0.176	0.162	0.156	0.080	0.069	0.197	0.187	0.197	0.205
*b* = 0.3	0.301	0.244	0.242	0.116	0.090	0.333	0.333	0.308	0.313
*b* = 0.4	0.437	0.345	0.337	0.169	0.119	0.477	0.443	0.440	0.438
*b* = 0.5	0.579	0.449	0.437	0.233	0.151	0.615	0.578	0.558	0.555
*b* = 0.6	0.702	0.546	0.530	0.316	0.186	0.739	0.700	0.679	0.665
*b* = 0.7	0.801	0.634	0.617	0.400	0.228	0.828	0.803	0.780	0.768
*b* = 0.8	0.874	0.708	0.693	0.486	0.277	0.893	0.877	0.858	0.849
*b* = 0.9	0.923	0.775	0.759	0.565	0.326	0.939	0.927	0.910	0.905
*b* = 1.0	0.954	0.827	0.812	0.643	0.364	0.962	0.960	0.947	0.941

The optimal choice of *K* will undoubtedly depend on the underlying correlation structure; however, the results of this section do suggest a rule of thumb for choosing *K*. First, for all values of *n* considered, smaller values of *K* yielded higher power, for both positive and negative network autocorrelation, and specifically *K* = 1 yielded the highest power. In addition, as discussed in S1 Appendix and shown in S8 Fig, convergence to the asymptotic chi-squared distribution was faster for smaller values of *K*. Second, in the presence of negative network autocorrelation, *K* = 1 performed significantly better than the other values of *K* considered. Taken together, the results suggest that if little is known about the underlying correlation structure, smaller values of *K* should be preferred, and specifically *K* = 1.

Although the network Ljung-Box test performed only marginally better than the Moran’s *I* with weight matrix *W*_1_, we note there are several advantages to using the proposed test. First, it is simple to implement and has a well-defined asymptotic null distribution, and this latter point is particularly desirable for extremely large networks for which non-parametric randomization tests could be computationally intensive. Second, its relation to the well known Ljung-Box test for serial autocorrelation provides a familiar framework for researchers. Third, as discussed in S1 Appendix, convergence to the asymptotic distribution occurred very quickly for *K* = 1. Hence, the network Ljung-Box test could also be preferable for very small networks. Finally, while our numerical results showed the performance of Moran’s *I* was sensitive to the choice of weight matrix, the network Ljung-Box test was comparatively more robust to the choice of *K*, particularly in the presence of positive network autocorrelation. Hence, unless the underlying correlation structure is known and strongly suggests a particular weight matrix be used, the network Ljung-Box test might be a more reliable test than Moran’s *I*.

## Example: Spatial autoregressive models for COVID-19 cases

In this section, we present an example application of the proposed network Ljung-Box test to COVID-19 case figures using data downloaded from the COVID-19 Data Repository by the Center for Systems Science and Engineering at Johns Hopkins University, which original sourced the data from the New York State Department of Health. In particular, we apply the test to examine the goodness-of-fit of spatial autoregressive models, by testing whether the model-based residuals are autocorrelated. We fit spatial autoregressive models to the natural logarithms of cumulative COVID-19 case figures of the 62 counties in New York state, as of February 1st, 2021, and test whether the resulting residual terms exhibit any spatial autocorrelation. The results are similar if we choose a different date after June 2020, when the epidemic has spread to the rural areas of the state. In the supplementary material, to demonstrate the ease at which our test can be extended to include a temporal component, we also fit spatial-temporal autoregressive models to the natural logarithms of monthly confirmed COVID-19 case figures of the 62 counties in New York state, over the period from June 2020 to January 2021, and test for the presence of any residual spatial-temporal correlation.

Let *C*_*i*_ denote the number of confirmed COVID-19 case numbers of county *i*, for *i* = 1, 2, …, 62. We construct a graph by connecting two counties with an edge if they directly border each other. We also define the index sets Ξ_*i*,*k*_, for *i* = 1, 2, …, 62, as the set of counties that are exactly *k* distance from county *i*, i.e., all counties *j* such that *d*(*v*_*i*_, *v*_*j*_) = *k*. Then the spatial AR(*p*) model that we consider is defined by

$$\begin{array}{c} \text{log}\;C_{i}=\alpha +\sum_{l=1}^{p}\beta_{l}\frac{\sum_{j\in \Xi_{i,l}}\text{log}\;C_{j}}{|\Xi_{i,l}|}+\xi_{i},\;i=1,2,\cdots ,62, \end{array}$$ (10)

where $\xi_{i}∼N\left(0,\sigma^{2}\right)$ are random noise terms and *α*, *β*_1_, …, *β*_*p*_ > 0. Note that we apply a logarithmic transformation to confirmed case numbers owing to the geometric nature of disease transmission. Let $T^{\left(l\right)}=[t_{ij}^{\left(l\right)}]$ denote the matrix with elements defined by

$$\begin{array}{c} t_{ij}^{\left(l\right)}=\frac{1}{|\Xi_{i,l}|}1\{j\in \Xi_{i,l}\}, \end{array}$$

where **1**{⋅} denotes the indicator function. Then the model (10) can be expressed in matrix form as

$$\begin{array}{c} \vec{C}=\vec{\alpha }+\sum_{l=1}^{p}\beta_{l}T^{\left(l\right)}\vec{C}+\vec{\xi }, \end{array}$$ (11)

where $\vec{C}={\left[\text{log}\;C_{1},\ldots ,\text{log}\;C_{62}\right]}^{⊤}$, $\vec{\xi }∼N\left(0,\sigma^{2}I_{62}\right)$ and $\vec{\alpha }={\left[\alpha ,\ldots ,\alpha \right]}^{⊤}$. Then we have

$$\vec{C}={\left(I_{62}-\sum_{l=1}^{p}\beta_{l}T^{\left(l\right)}\right)}^{-1}(\vec{\alpha }+\vec{\xi }),$$

so that the likelihood is multivariate normal. Maximum likelihood estimates are obtained numerically using the Nelder-Mead method.

The fitted models are given in Table 5. We consider the spatial AR(0), AR(1), AR(2) and AR(3) models in our analysis, where the AR(0) model, including only the parameter *α*, does not attempt to account for spatial autocorrelation. Using the fitted models, the residual terms are then reconstructed as

$$\begin{array}{c} {\hat{\xi }}_{i}=\text{log}\;C_{i}-\hat{\alpha }-\sum_{l=1}^{p}{\hat{\beta }}_{l}\frac{\sum_{j\in \Xi_{i,l}}\text{log}\;C_{j}}{|\Xi_{i,l}|},\;i=1,2,\cdots ,62. \end{array}$$

The frequency distributions for the residual terms under each of the spatial autoregressive models are given in S4–S7 Figs of the supplementary material. The sample mean of the error terms under each case is approximately 0. A skew is clearly evident in the distribution of the AR(0) random noise terms, while the random noise terms of the AR(1), AR(2) and AR(3) models are approximately symmetric, albeit with a slight skew, and approximately bell-curve shaped. We calculate the ratio $\hat{\lambda }=\bar{X^{4}}/{\left(\bar{X^{2}}\right)}^{2}$, where $\bar{X^{2}}$ and $\bar{X^{4}}$ are the sample second and fourth moments, respectively. This gives ${\hat{\lambda }}_{0}=2.75$, ${\hat{\lambda }}_{1}=2.96$, ${\hat{\lambda }}_{2}=3.12$ and ${\hat{\lambda }}_{3}=3.17$ for the spatial AR(0), AR(1), AR(2) and AR(3) models, respectively, which we use when calculating the test statistic *Q*(*K*) defined by (6). For the AR(1), AR(2) and AR(3) models, the approximately bell-curved histograms in S5–S7 Figs of the supplementary material, combined with ${\hat{\lambda }}_{i}$ being close to 3, appears to validate the assumption of Gaussian errors.

**Table 5:** Fitted spatial autoregressive models. Fitted spatial AR(*p*) models to logarithms of confirmed COVID-19 cases in New York counties.

Fitted Autoregressive Models						
	$\hat{\alpha }$	${\hat{\beta }}_{1}$	${\hat{\beta }}_{2}$	${\hat{\beta }}_{3}$	$\hat{\sigma }$	AIC
AR(0)	8.798	–	–	–	1.505	230.639
AR(1)	2.626	0.702	–	–	1.029	194.531
AR(2)	1.902	0.497	0.289	–	1.022	193.556
AR(3)	1.027	0.457	0.084	0.348	0.991	191.841

We conduct the network Ljung-Box test on the reconstructed error terms for each of the spatial autoregressive models, and consider the spatial lags *K* = 1, 2, 3, 4, 5, 6. The observed test statistics and their corresponding *p*-values are given in Table 6. For the AR(0) model, we find that the network Ljung-Box test strongly rejects the null hypothesis that the error terms are spatially independent at the 5% significance level for all spatial lags *K* considered, and thus conclude that spatial correlation indeed exists in county-level COVID-19 case figures. In other words, counties within close geographic proximity experienced similar severity outbreaks, suggesting that cross-county migration might have played a role, along with other shared factors between neighboring counties. In contrast, for the AR(1), AR(2) and AR(3) models, we find that spatial independence is accepted at the 5% significance level for all lags *K* = 1, 2, 3, 4, 5, 6. This suggests that spatial autocorrelation in the data no longer persists after fitting spatial autoregressive models; indeed, referring to the power analysis results given in Table 2, the simulations showed a high statistical power for positive spatial autocorrelation *b* = 0.5 at *n* = 50, suggesting that acceptance is not necessarily due to having a small sample size of 62 counties. According to AIC, the best fitting model is found to be the AR(3) model, while the worst fitting, unsurprisingly, is the AR(0) model that does not account for any spatial autocorrelation.

**Table 6:** Goodness-of-fit for fitted spatial autoregressive models. Obtained *p*-values for error terms of the spatial AR(0), AR(1), AR(2) and AR(3) models (*n* = 62).

	AR(0)		AR(1)		AR(2)		AR(3)	
Spatial lags	*Q*(*K*)	*p*-value	*Q*(*K*)	*p*-value	*Q*(*K*)	*p*-value	*Q*(*K*)	*p*-value
*K* = 1	37.043	*p* < 0.001	0.706	0.401	0.095	0.758	0.006	0.938
*K* = 2	54.616	*p* < 0.001	0.738	0.691	2.120	0.347	0.551	0.759
*K* = 3	68.956	*p* < 0.001	4.290	0.232	5.601	0.133	1.171	0.760
*K* = 4	71.117	*p* < 0.001	4.423	0.352	5.625	0.229	1.179	0.882
*K* = 5	71.348	*p* < 0.001	4.456	0.486	5.639	0.343	1.450	0.919
*K* = 6	74.773	*p* < 0.001	4.505	0.609	5.670	0.461	1.728	0.943

## Conclusion

In this paper, we have outlined a simple portmanteau test for network autocorrelation supported by a derivation of the asymptotic distribution of the test statistic which turns out to be chi-squared provided certain mild conditions hold. Detailed simulations are given using a real network graph which both confirm the asymptotic distribution and also demonstrate the rate of convergence. Specifically, we find that convergence is achieved faster for a smaller number of network lags due to the assumption of asymptotic independence underpinning the test. Power analysis simulations are also given which demonstrate a significant improvement in power using the proposed network Ljung-Box test compared to the expected similarity and similarity entropy tests proposed by Farber et al. [19], and also a higher power than the widely used Moran’s *I* test, the performance of which depends critically on a chosen weight matrix. This improvement in power is observed to hold across a wide variety of sample sizes, and for varying degrees and signs of network autocorrelation. Interestingly, it is found that the statistical power of the network Ljung-Box test with a large number of network lags depends strongly on the sign of the network autocorrelation, such that a small number of lags should be included when negative autocorrelation is suspected. Nevertheless, the power simulations demonstrate the network Ljung-Box test can achieve high power for both positive and negative network autocorrelation. Two important yet straightforward extensions of the proposed test are also discussed; namely, the inclusion of a temporal component and the application of the network Ljung-Box test to weighted graphs. Finally, an example application of the test to COVID-19 case figures is given, which shows that the fitted spatial autoregressive models are able to adequately account for the effect of transmission due to population flow between neighboring counties.

## Supporting information

S1 Appendix Convergence of the null distribution. (PDF)

S2 Appendix A spatial-temporal autoregressive model for COVID-19 case figures. (PDF)

S3 Appendix Proofs. (PDF)

S1 Fig Type-I error rates. Type-I error rates of the network Ljung-Box test for network lags *K* = 1, 2, 3, 4 compared to expected similarity, similarity entropy and Moran’s *I* tests.
(TIFF)

S2 Fig Statistical power for positive network autocorrelation. Statistical power of the network Ljung-Box test for network lags *K* = 1, 2, 3, 4 compared to expected similarity, similarity entropy and Moran’s *I* tests for fixed *b* = 0.5.
(TIFF)

S3 Fig Statistical power for negative network autocorrelation. Statistical power of the network Ljung-Box test for network lags *K* = 1, 2, 3, 4 compared to expected similarity, similarity entropy and Moran’s *I* tests for fixed *b* = −0.5.
(TIFF)

S4 Fig Distribution of noise for spatial AR(0) model. Density histograms of the error terms for the spatial AR(0) model.
(TIFF)

S5 Fig Distribution of noise for spatial AR(1) model. Density histograms of the error terms for the spatial AR(1) model.
(TIFF)

S6 Fig Distribution of noise for spatial AR(2) model. Density histograms of the error terms for the spatial AR(2) model.
(TIFF)

S7 Fig Distribution of noise for spatial AR(3) model. Density histograms of the error terms for the spatial AR(3) model.
(TIFF)

S8 FigConvergence of *Q*(*K*) to the chi-squared $\chi_{K}^{2}$ distribution.The KS distances between simulated distributions of *Q*(*K*) and the asymptotic chi-squared distribution for different values of *n*.(TIFF)

S9 Fig Noise distribution for *AR*(1, 1) model. Density histogram of the reconstructed error terms for the spatial-temporal AR(1,1) model.
(TIFF)

S10 Fig Noise distribution for *AR*(2, 1) model. Density histogram of the reconstructed error terms for the spatial-temporal AR(2,1) model.
(TIFF)

S11 Fig Volatility of AR(1,1) model. Monthly sample standard deviations of the reconstructed error terms for the spatial-temporal AR(1,1) model.
(TIFF)

S12 Fig Volatility of AR(2,1) model. Monthly sample standard deviations of the reconstructed error terms for the spatial-temporal AR(2,1) model.
(TIFF)

S1 TableKolmogorov-Smirnov distances for simulated test statistic distributions.KS distances between simulated *Q*(*K*) and $\chi_{K}^{2}$ distributions for different values of *n* and *K* using the immuno dataset.(PDF)

S2 Table Fitted spatial-temporal autoregressive models. Fitted spatial-temporal AR(*p*,1) models to logarithms of confirmed COVID-19 cases in New York counties.
(PDF)

S3 Table Obtained *p*-values for spatial-temporal independence. Obtained *p*-values for error terms of the spatial-temporal AR(*p*,1) model (*n* = 434).
(PDF)

S1 Data (ZIP)
