# Supplementary material for: Testing network autocorrelation without replicates
Source: PLoS One. 2022 Nov 3;17(11):e0275532. doi: 10.1371/journal.pone.0275532 (PMC9632870; doi:10.1371/journal.pone.0275532)
Supplement: S1 Appendix — (PDF) [file pone.0275532.s001.pdf]

S1 Appendix: Convergence of the null distribution

To study the convergence of the test statistic to the asymptotic chi-squared distribution, for each n, a test statistic distribution is obtained by generating $20,000$ such sets of associated random variables and calculating the test statistic $Q(K)$ for each set according to the network structure of the subgraph G_n^I. These test statistic distributions are then compared to the chi-squared distribution χ^2_K using the Kolmogorov-Smirnov (KS) distance. The results are summarized in S1 Table, while S8 Fig plots the KS distance against n for each value of K.

The results show that the test statistic $Q(K)$ appears to converge to χ^2_K for all values of K considered, with the observed KS distances decreasing as we increase the number of vertices n. Interestingly, for a fixed number of vertices, we find that the KS distance tends to increase with K, the number of network lags. In other words, *ceteris paribus*, convergence to the asymptotic chi-squared distribution is faster when the test statistic includes fewer network lags. One explanation for this behavior is that the summands of $Q(K)$ are only asymptotically independent; although they are uncorrelated, they are only asymptotically normal, and therefore only independent at the limit. Thus, for small values of n this dependence may be non-negligible and therefore the chi-squared construction, i.e., the sum of squares of independent standard normal random variables, may not hold. Thus, for small sample sizes, when implementing the proposed network Ljung-Box test, the number of network lags included should be kept small in order to avoid significant deviations from the asymptotic chi-squared distribution. Nevertheless, S8 Fig demonstrates clearly that the test statistic indeed converges to χ^2_K for all network lags K considered.
