# Supplementary material for: Testing network autocorrelation without replicates
Source: PLoS One. 2022 Nov 3;17(11):e0275532. doi: 10.1371/journal.pone.0275532 (PMC9632870; doi:10.1371/journal.pone.0275532)
Supplement: S2 Appendix — (PDF) [file pone.0275532.s002.pdf]

S2 Appendix: A spatial-temporal autoregressive model for COVID-19 case figures

We consider a spatial-temporal autoregressive model for monthly COVID-19 case figures of New York counties. This example is given to demonstrate how testing for temporal and network autocorrelation is functionally equivalent in our extended graphical framework. To this end, let $C_i(t)$ denote the cumulative number of confirmed COVID-19 case numbers of county i in month t, for $i = 1, 2, \ldots, 62$ and month $i = 1, 2, \ldots, 8$, where we consider the months between June 2020 and January 2021, inclusive. Shorter time periods are not considered to avoid periods where counties report no cases. We then consider a spatial-temporal autoregressive AR(p,1) model of the form

$$\log\left(\frac{C_i(t)}{C_i(t-1)}\right) = \alpha + \sum_{l=1}^{p} \beta_l \sum_{j \in \Xi_{i,l}} \frac{1}{|\Xi_{i,l}|} \log\left(\frac{C_j(t)}{C_j(t-1)}\right) + \gamma \log\left(\frac{C_i(t-1)}{C_i(t-2)}\right) + \xi_i(t),$$

for $t = 2, 3, \ldots, 8$ and $i = 1, 2, \ldots, 62$, so that the proportional increase in the number of confirmed cases in county i in month t depends on the number of confirmed cases in county i in month $t-1$, along with the average number of cases in neighboring counties in month t, where the latter represents the spillover effect of cross-county migration. The parameters $\alpha, \beta_1, \ldots, \beta_p, \gamma$ are again estimated using maximum likelihood, and the random noise terms reconstructed for all i and t. The estimates obtained are given in S2 Table. The density histograms of the error terms, across all counties and months, are given in S9-S10 Figs and both exhibit strong skews demonstrating a pronounced lack of normality. We estimate λ using the sample second and fourth moments as $\hat{\lambda}_{1,1} = 4.897$, $\hat{\lambda}_{2,1} = 5.208$, far from $\lambda = 3$ of the normal distribution. The p-values for different lags $K = 1, 2, 3, 4, 5, 6$ are given in S3 Table, for which the null hypothesis of spatial-temporal independence of monthly COVID-19 county cases is strongly rejected at the 5% significance level for all lags.

In fact, to see why the test is rejected so strongly for the spatial-temporal autoregressive models, but not for the spatial autoregressive models, we can examine the distribution of the residuals on a monthly basis. Specifically, to test for the presence of spatial-temporal autocorrelation, the assumptions underlying the network Ljung-Box test require the residuals to be stationary over time. However, after fitting the AR(1,1) and AR(2,1) models we observe changing levels of volatility, despite standardizing the residuals by considering the logarithm of proportional case figures, as shown in S11-S12 Figs. It is interesting to note that under both models volatility starts low, peaks around October, before decreasing thereafter.
