# Supplementary material for: Testing network autocorrelation without replicates
Source: PLoS One. 2022 Nov 3;17(11):e0275532. doi: 10.1371/journal.pone.0275532 (PMC9632870; doi:10.1371/journal.pone.0275532)
Supplement: S3 Appendix — (PDF) [file pone.0275532.s003.pdf]

S3 Appendix: Proofs

The following are proofs to the theoretical results presented in the main manuscript.

Proof of Proposition 3:

We can rewrite S_k^n as

$$S_k^n = \sum_{(i,j)\in U_k^n} X_i X_j = \sum_{i\in\Gamma_k^n} \sum_{j\in\Xi_{i,k}^n} X_i X_j.$$

Denote

$$Y_i^n := \sum_{j\in\Xi_{i,k}^n} X_i X_j, \quad Y_i := \sum_{j\in\Xi_{i,k}} X_i X_j.$$

Obviously, if $i \notin \Gamma_k^n$, then $\Xi_{i,k}^n = \emptyset$ and so $Y_i^n = 0$. We desire to use Theorem 1 of [?] to establish the CLT. To achieve this, we need to verify the physical dependence measure for Y_i, $i \in \mathbb{Z}$, over the whole domain. We can then calculate that

$$\begin{aligned}
\delta_{i,p/2}(Y) &= \| \sum_{j\in\Xi_{i,k}} X_i X_j - \sum_{j\in\Xi_{i,k}} X_i^* X_j^* \|_{p/2} \\
&\le \| \sum_{j\in\Xi_{i,k}} X_i X_j - \sum_{j\in\Xi_{i,k}} X_i X_j^* \|_{p/2} + \| \sum_{j\in\Xi_{i,k}} X_i X_j^* - \sum_{j\in\Xi_{i,k}} X_i^* X_j^* \|_{p/2} \\
&\le \|X_i\|_p \sum_{j\in\Xi_{i,k}} \|X_j - X_j^*\|_p + \sum_{j\in\Xi_{i,k}} \|X_i - X_i^*\|_p \|X_j^*\|_p \\
&\le \sum_{j\in\Xi_{i,k}} \|X_0\|_p (\delta_{j,p} + \delta_{i,p}).
\end{aligned}$$

Hence,

$$\Delta_{p/2}^Y = \sum_{i\in\mathbb{Z}} \delta_{i,p/2}(Y) \le \sum_{i\in\Gamma_k} \sum_{j\in\Xi_{i,k}} \|X_0\|_p (\delta_{j,p} + \delta_{i,p}) = \|X_0\|_p \sum_{(i,j)\in U_k} (\delta_{i,p} + \delta_{j,p}).$$

As a result, if condition (i) holds, then we have $\Delta_2^Y < \infty$ and the CLT holds for $\sum_{(i,j)\in U_k^n} X_i X_j / |U_k^n|$ by Theorem 1 of [?] as long as conditions (ii) and (iii) are also satisfied.

Proof of Theorem 5:

To ease notation, denote $\mathbb{E}[X_i^2] = \sigma^2$, and define

$$C(k,n) := \frac{n(n+\lambda-1)}{|U_k^n|}, \quad x(k,n) := \sum_{(i,j)\in U_k^n} X_i^n X_j^n, \quad y(n) := \sum_{(i,i)\in U_0^n} (X_i^n)^2.$$

To complete the proof, it suffices to show that $\sqrt{C(k,n)} r_{k,n}$ are asymptotically i.i.d. standard normal random variables.

We begin by evaluating the moments of $y(n)$ and $x(k,n)$. By using the law of total expectation, it is immediately clear that $\mathbb{E}[y(n)] = n\sigma^2$. Moreover, we also have

$$\begin{aligned}
\mathbb{E}[y(n)^2] &= \mathbb{E}\left[\sum_{(i,i)\in U_0^n} (X_i^n)^4 + \sum_{(i,i)\in U_0^n} \sum_{j\neq i} (X_i^n)^2 (X_j^n)^2 \right] \\
&= n\lambda\sigma^4 + \sum_{(i,i)\in U_0^n} \sum_{j\neq i} \sigma^4 = n(n+\lambda-1)\sigma^4
\end{aligned}$$

where the second equality follows from the definition of λ. As a result, the variance of $y(n)$ is given by

$$Var(y(n)) = \mathbb{E}[y(n)^2] - \mathbb{E}[y(n)]^2 = n(\lambda - 1)\sigma^4.$$

Therefore, as $n \to \infty$ we have $Var\left(\frac{y(n)}{n}\right) \to 0$, and hence

$$\frac{y(n)}{n} \xrightarrow{p} \mathbb{E}[X_i^2] = \sigma^2.$$

Next, we likewise evaluate the moments of $x(k,n)$. By the assumption of independence, and the fact the variables are centered, we have

$$\mathbb{E}[x(k,n)] = \sum_{(i,j)\in U_k^n} \mathbb{E}[X_i^n X_j^n] = \sum_{(i,j)\in U_k^n} \mathbb{E}[X_i^n]\mathbb{E}[X_j^n] = 0,$$

and similarly

$$\mathbb{E}[x(k,n)^2] = \sum_{(i,j)\in U_k^n} \sum_{(m,l)\in U_k^n} \mathbb{E}[X_i^n X_j^n X_m^n X_l^n] = \sum_{(i,j)\in U_k^n} \mathbb{E}[(X_i^n)^2 (X_j^n)^2] = |U_k^n|\sigma^4$$

where the second inequality follows from the fact that if $(i,j) \neq (m,l)$, then the independence and zero-mean assumptions imply $\mathbb{E}[X_i^n X_j^n X_m^n X_l^n] = 0$. As a result, it follows that as $n \to \infty$ we have

$$\mathbb{E}\left[\left(\frac{x(k,n)^2}{n}\right)^2\right] \to 0.$$

On the other hand, for $k \neq l$, we have

$$Cov\big(x(k,n), x(l,n)\big) = \mathbb{E}\left[\sum_{(i,j)\in U_k^n} X_i^n X_j^n \sum_{(m,h)\in U_l^n} X_m^n X_h^n\right] = 0$$

since it is impossible for $(i,j) = (m,h)$ if $(i,j) \in U_k^n$ and $(m,h) \in U_l^n$, and hence we have $\mathbb{E}[X_i^n X_j^n X_m^n X_h^n] = 0$ for all summands. An application of Slutsky's theorem then yields

$$\mathbb{E}[r_{k,n}] = \mathbb{E}\left[\frac{x(k,n)}{y(n)}\right] = \mathbb{E}\left[\frac{\frac{1}{n}x(k,n)}{\frac{1}{n}y(n)}\right] \to 0$$

and

$$\mathbb{E}[r_{k,n} r_{l,n}] = \mathbb{E}\left[\frac{\frac{1}{n}x(k,n)\frac{1}{n}x(l,n)}{\frac{1}{n^2}y(n)^2}\right] \to 0$$

as $n \to \infty$. Hence $r_{k,n}$ and $r_{l,n}$ are asymptotically uncorrelated for $k \neq l$. Further, if $r_{k,n}$ and $r_{l,n}$ are, after rescaling, asymptotically normal, then they will be asymptotically independent. We also have

$$\mathbb{E}[C(k,n) r_{k,n}^2] = \frac{n + \lambda - 1}{n} \mathbb{E}\left[\frac{x(k,n)^2/|U_k|}{(y(n)/n)^2}\right] \to 1,$$

as $n \to \infty$. All that remains is to establish that $\sqrt{C(k,n)} r_{k,n}$ are indeed asymptotically normal using an applicable version of the CLT. Specifically, we need to show

$$\sqrt{\frac{n(n+\lambda-1)}{|U_k|}} \frac{\sum_{(i,j)\in U_k} X_i^n X_j^n}{\sum_{(i,i)\in U_0^n} (X_i^n)^2}$$

are asymptotically normal for $k = 1, 2, \ldots, K$. Note that we have

$$\frac{\sqrt{n(n+\lambda-1)}}{\sum_{(i,i)\in U_0^n}(X_i^n)^2} = \frac{n}{\sum_{(i,i)\in U_0^n}(X_i^n)^2}\sqrt{\frac{n(n+\lambda-1)}{n}} \xrightarrow{p} \frac{1}{\sigma^2}$$

and so we need only verify that

$$\frac{\sum_{(i,j)\in U_k^n} X_i^n X_j^n}{\sqrt{|U_k^n|}} \xrightarrow{d} N(0, \sigma^4). \tag{1}$$

To do this, we will apply Proposition 1, taking $g(\cdot)$ as the identity function, i.e., $X_i = g(\epsilon_i) = \epsilon_i$. This can be done since the random variables $X_1, X_2, \ldots, X_n$ are assumed to be i.i.d. Then clearly we have

$$\hat{\Delta}_k = \sum_{(i,j)\in U_k} (\delta_{i,4} + \delta_{j,4}) < \infty$$

using similar arguments as that after Corollary 1. Additionally, we have

$$\sigma_n^2 = \mathbb{E}\left[\left(\sum_{(i,j)\in U_k^n} X_i^n X_j^n\right)^2\right] = \sum_{(i,j)\in U_k^n} \mathbb{E}[(X_i^n)^2(X_j^n)^2] = |U_k^n|\sigma^4 \to \infty \quad as \quad n \to \infty.$$

It then immediately follows from Proposition 1 that

$$\frac{\sum_{(i,j)\in U_k^n} X_i^n X_j^n}{\sqrt{|U_k^n|}} \xrightarrow{d} N(0, \sigma^4),$$

completing the proof.
