# Supplementary figures and images for: Testing network autocorrelation without replicates
Source: PLoS One. 2022 Nov 3;17(11):e0275532. doi: 10.1371/journal.pone.0275532 (PMC9632870; doi:10.1371/journal.pone.0275532)

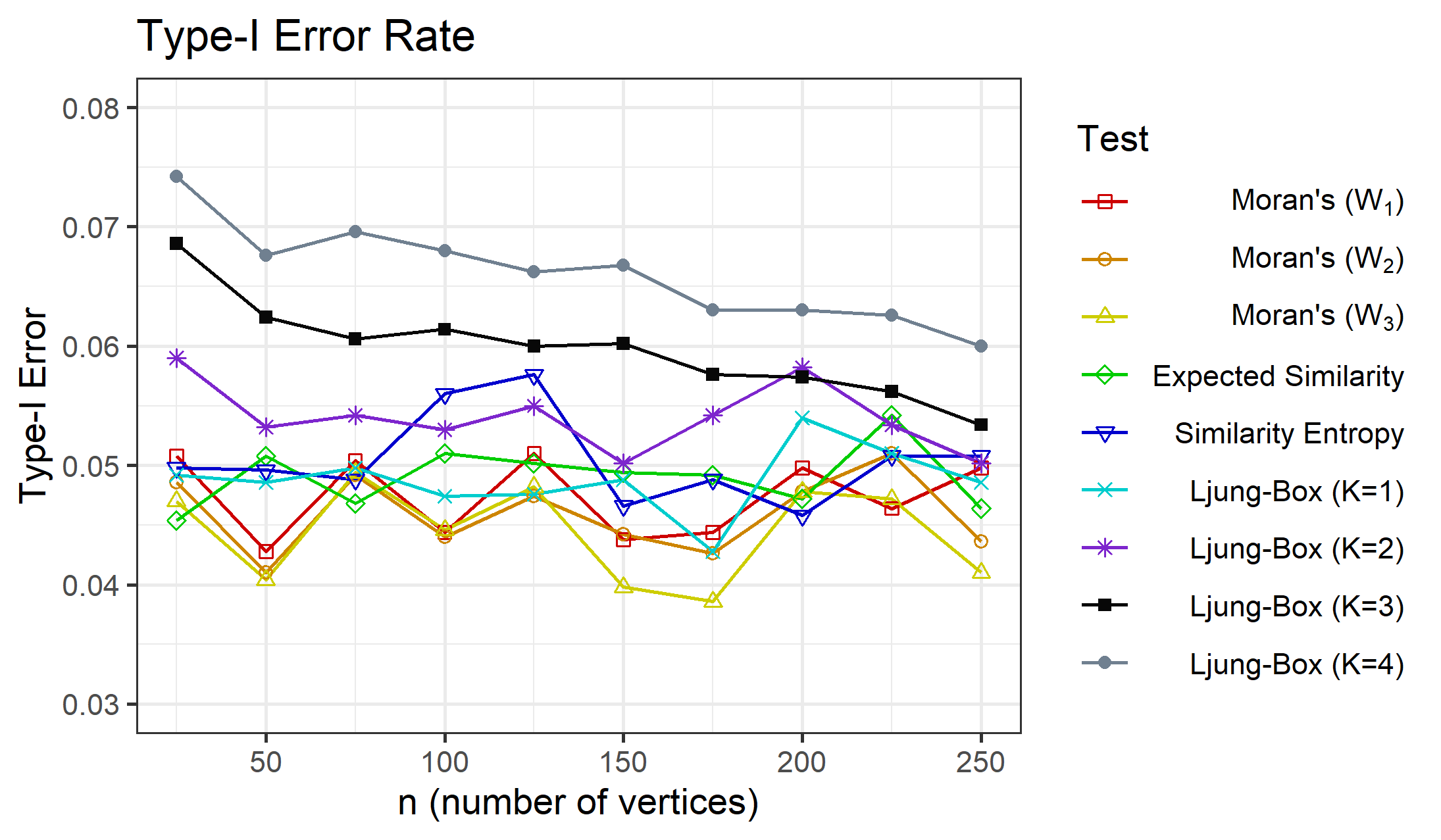

Supplement: S1 Fig — Type-I error rates of the network Ljung-Box test for network lags K = 1, 2, 3, 4 compared to expected similarity, similarity entropy and Moran’s I tests. (TIFF) [file pone.0275532.s004.tiff]

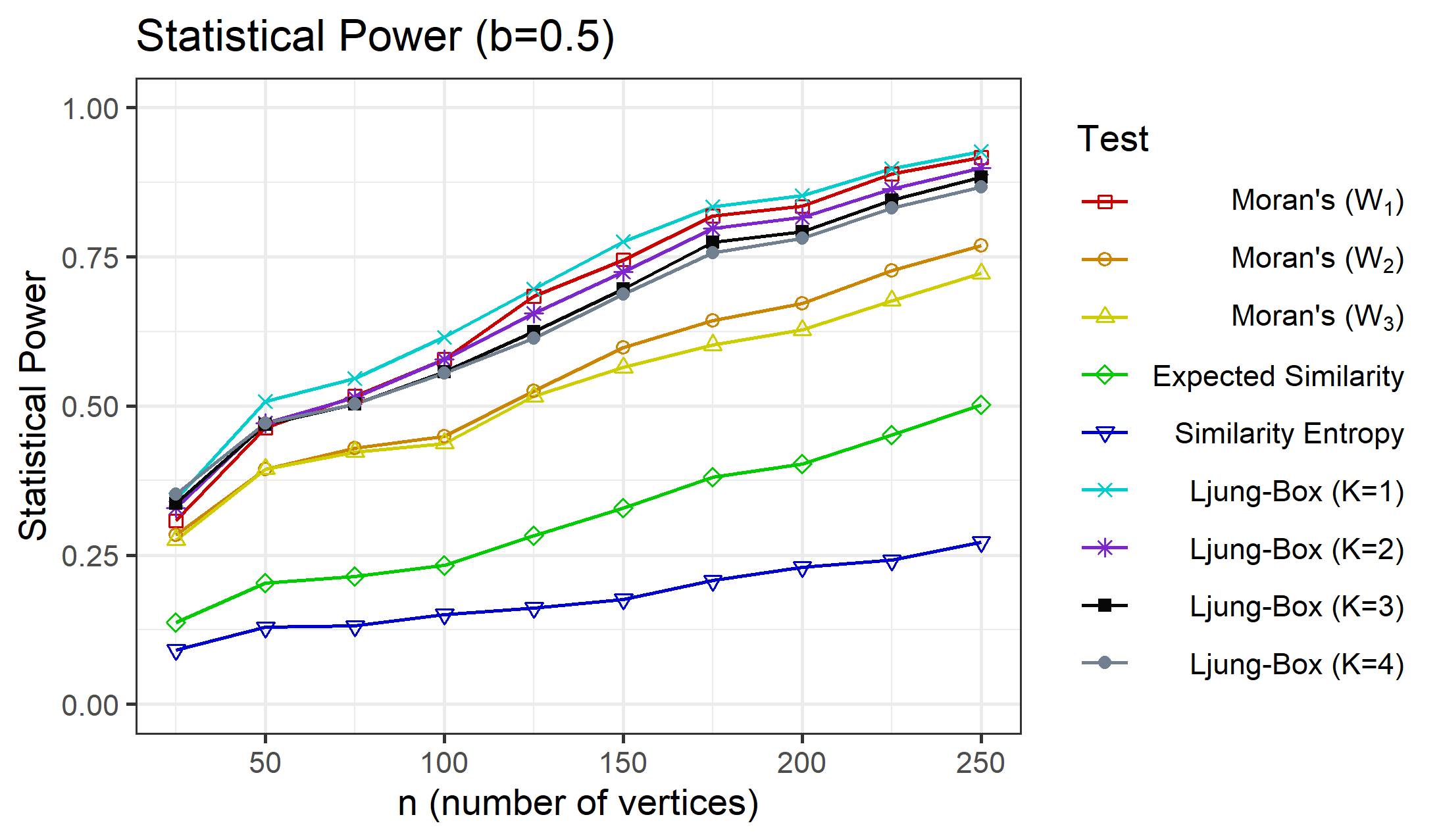

Supplement: S2 Fig — Statistical power of the network Ljung-Box test for network lags K = 1, 2, 3, 4 compared to expected similarity, similarity entropy and Moran’s I tests for fixed b = 0.5. (TIFF) [file pone.0275532.s005.tiff]

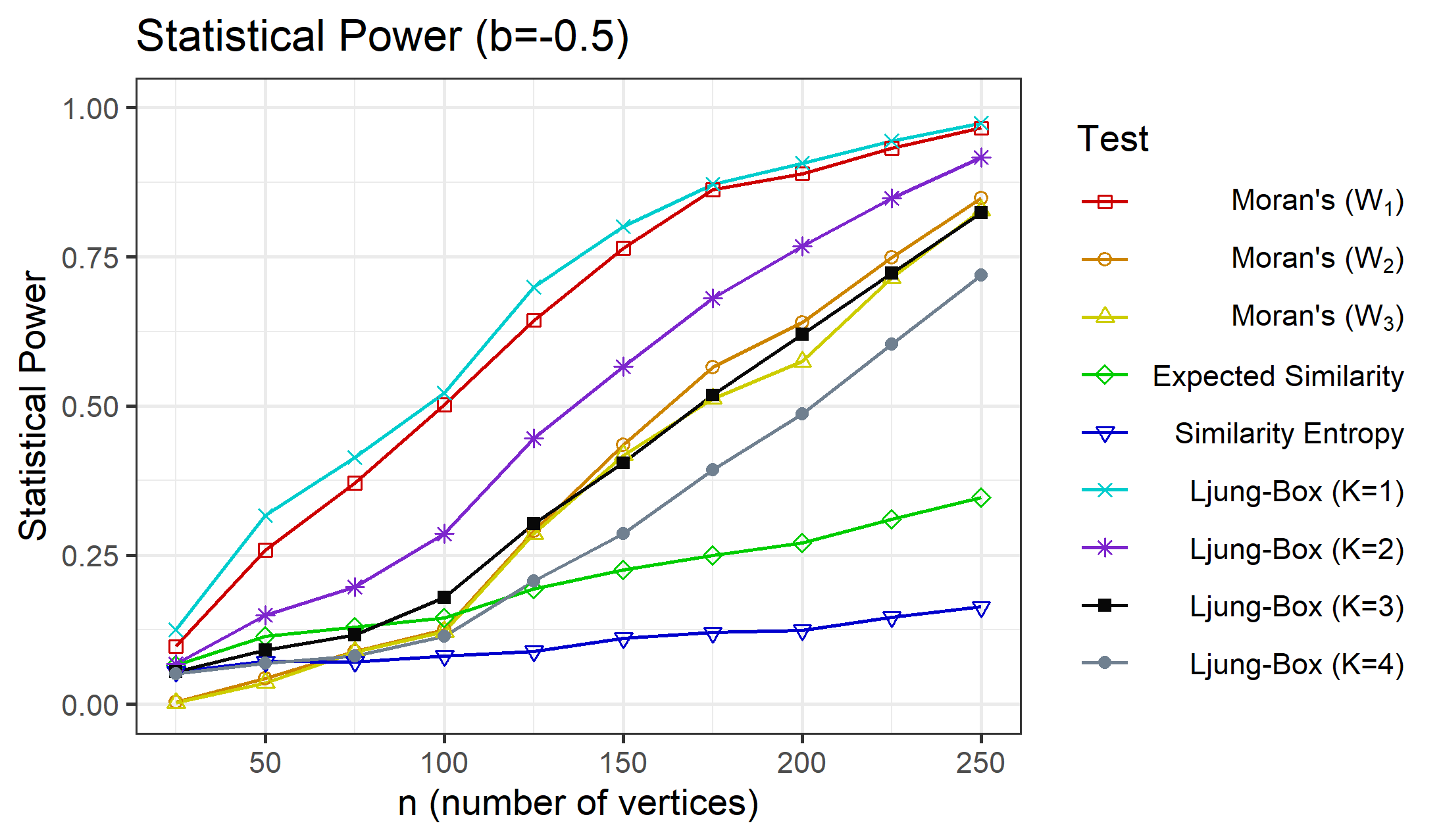

Supplement: S3 Fig — Statistical power of the network Ljung-Box test for network lags K = 1, 2, 3, 4 compared to expected similarity, similarity entropy and Moran’s I tests for fixed b = −0.5. (TIFF) [file pone.0275532.s006.tiff]

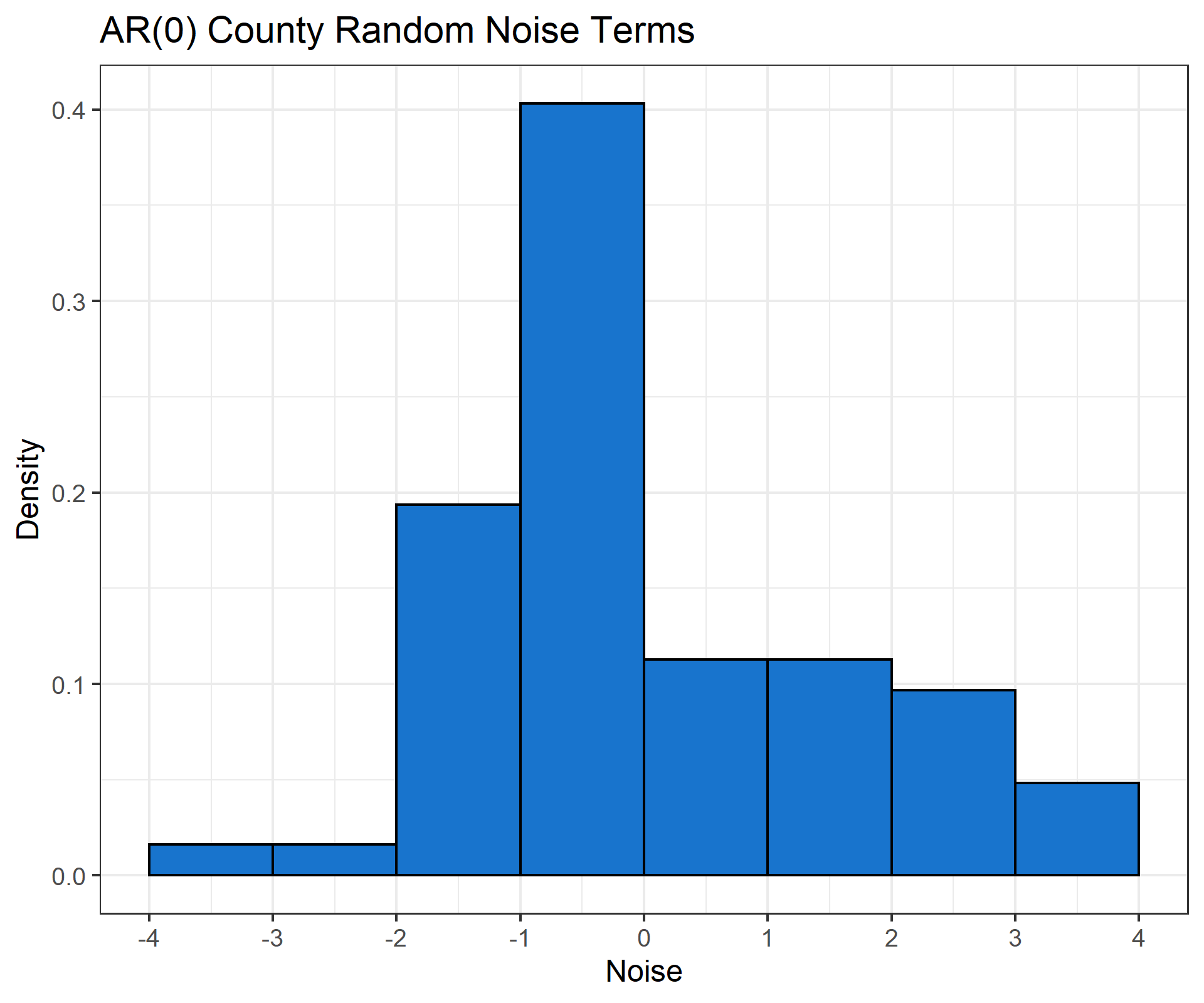

Supplement: S4 Fig — Density histograms of the error terms for the spatial AR(0) model. (TIFF) [file pone.0275532.s007.tiff]

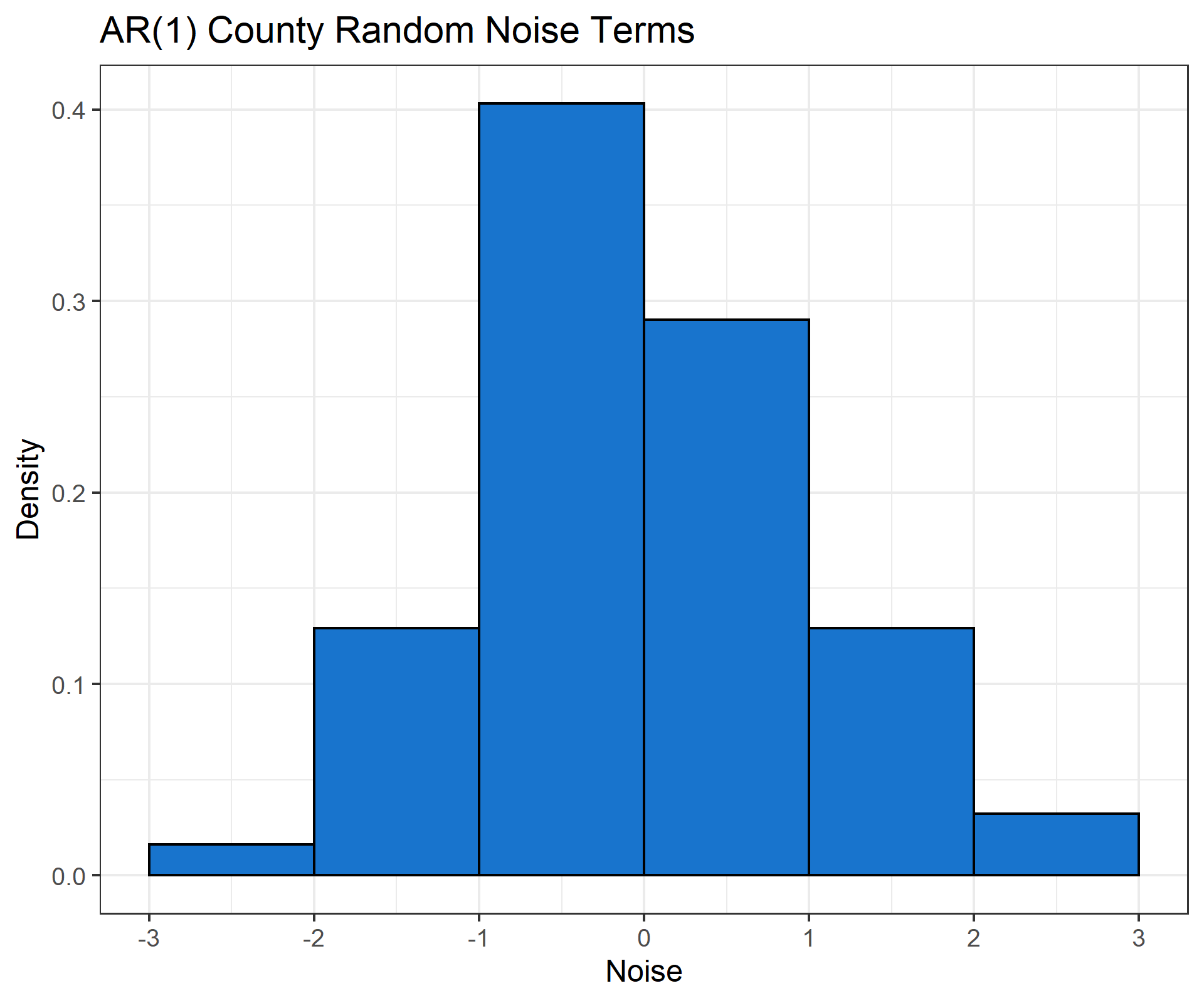

Supplement: S5 Fig — Density histograms of the error terms for the spatial AR(1) model. (TIFF) [file pone.0275532.s008.tiff]

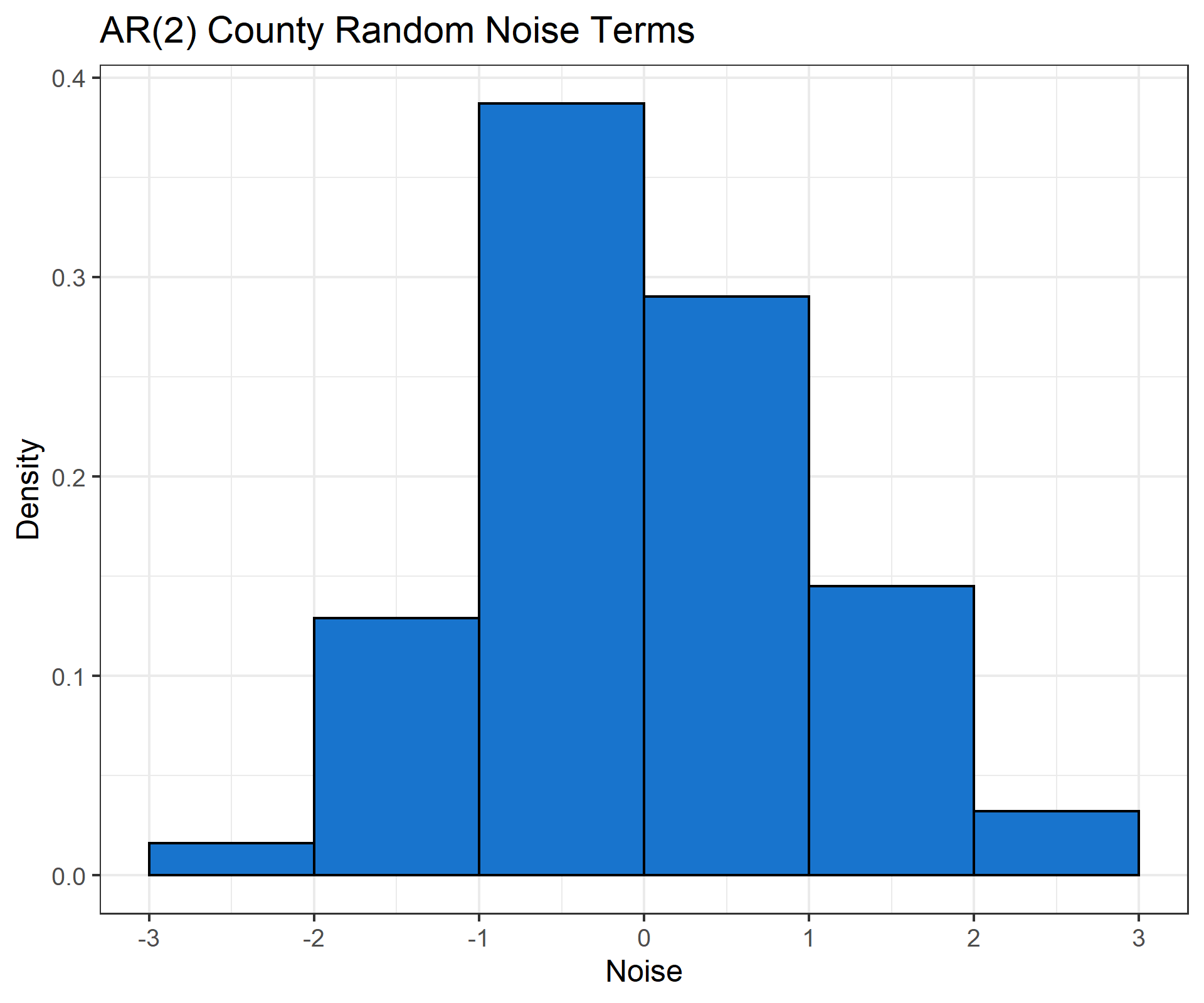

Supplement: S6 Fig — Density histograms of the error terms for the spatial AR(2) model. (TIFF) [file pone.0275532.s009.tiff]

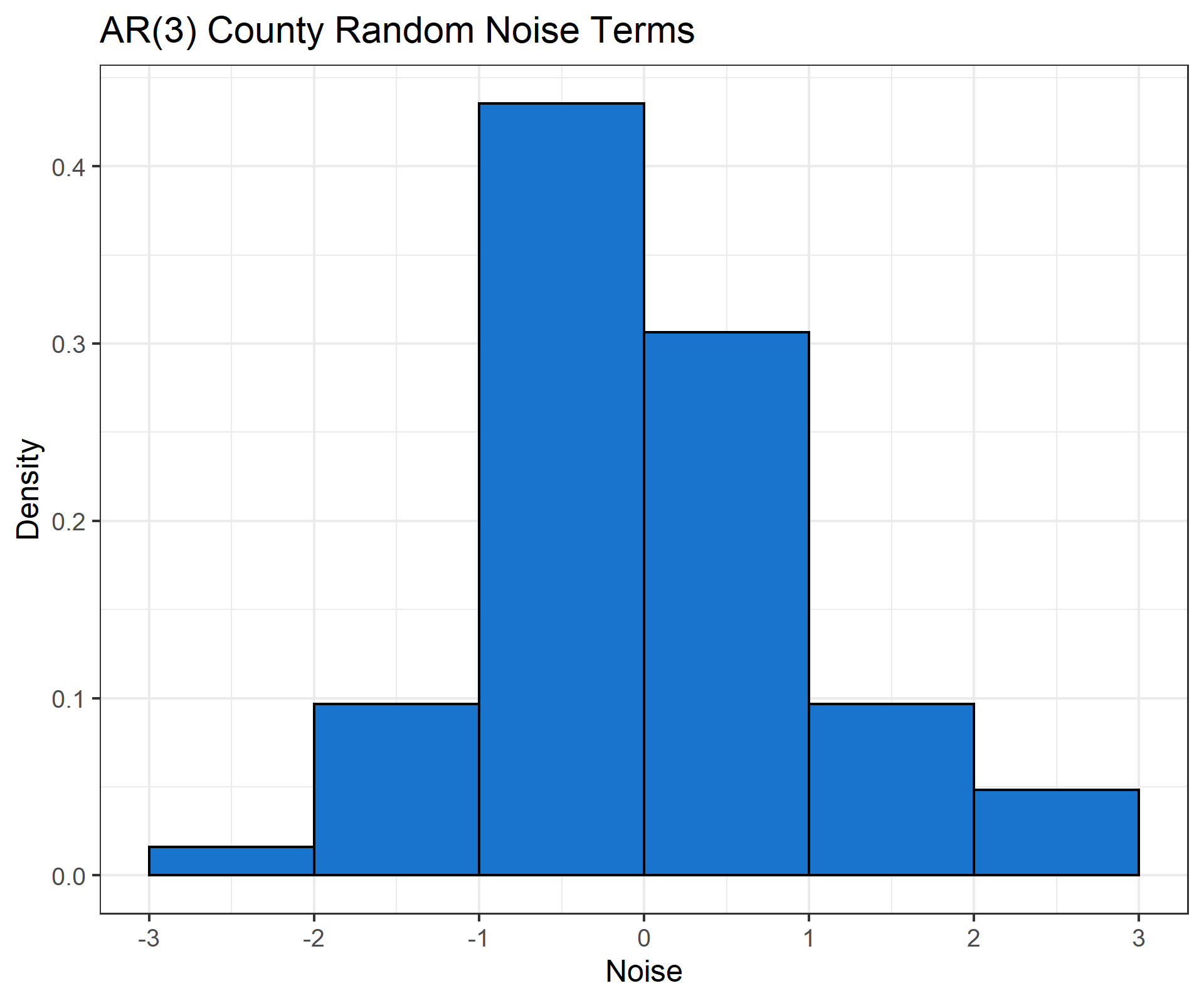

Supplement: S7 Fig — Density histograms of the error terms for the spatial AR(3) model. (TIFF) [file pone.0275532.s010.tiff]

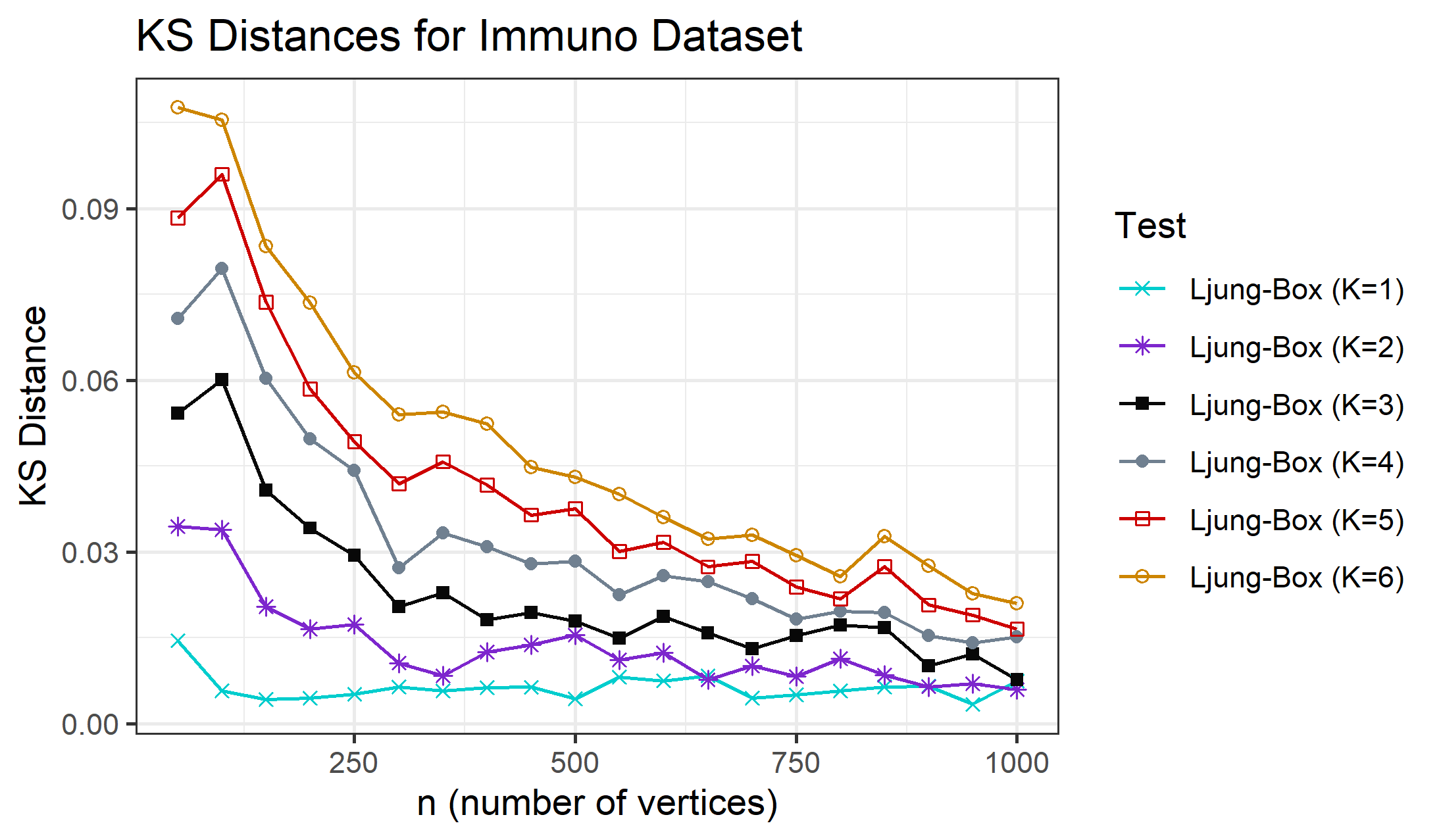

Supplement: S8 Fig — The KS distances between simulated distributions of Q(K) and the asymptotic chi-squared distribution for different values of n. (TIFF) [file pone.0275532.s011.tiff]

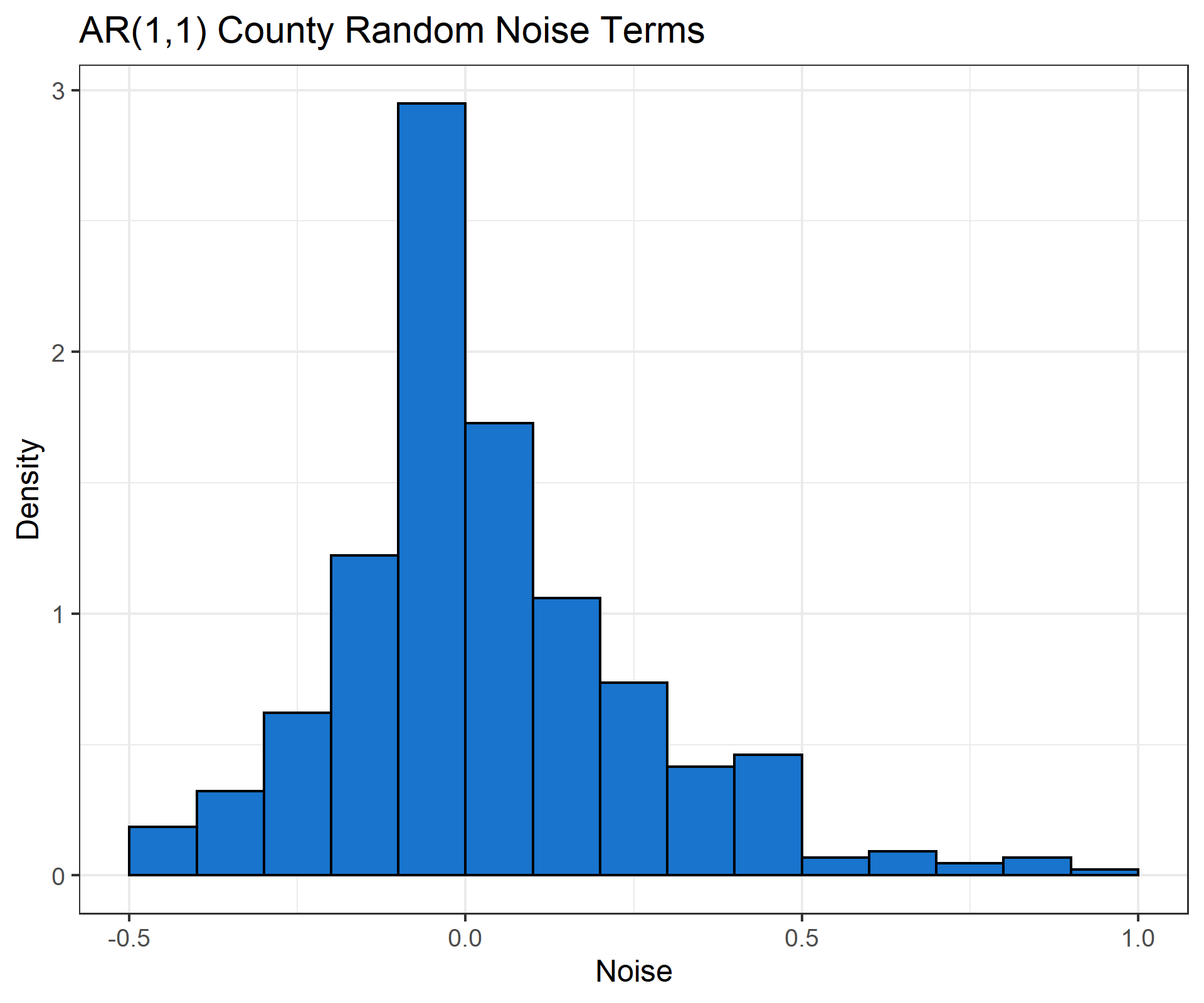

Supplement: S9 Fig — Density histogram of the reconstructed error terms for the spatial-temporal AR(1,1) model. (TIFF) [file pone.0275532.s012.tiff]

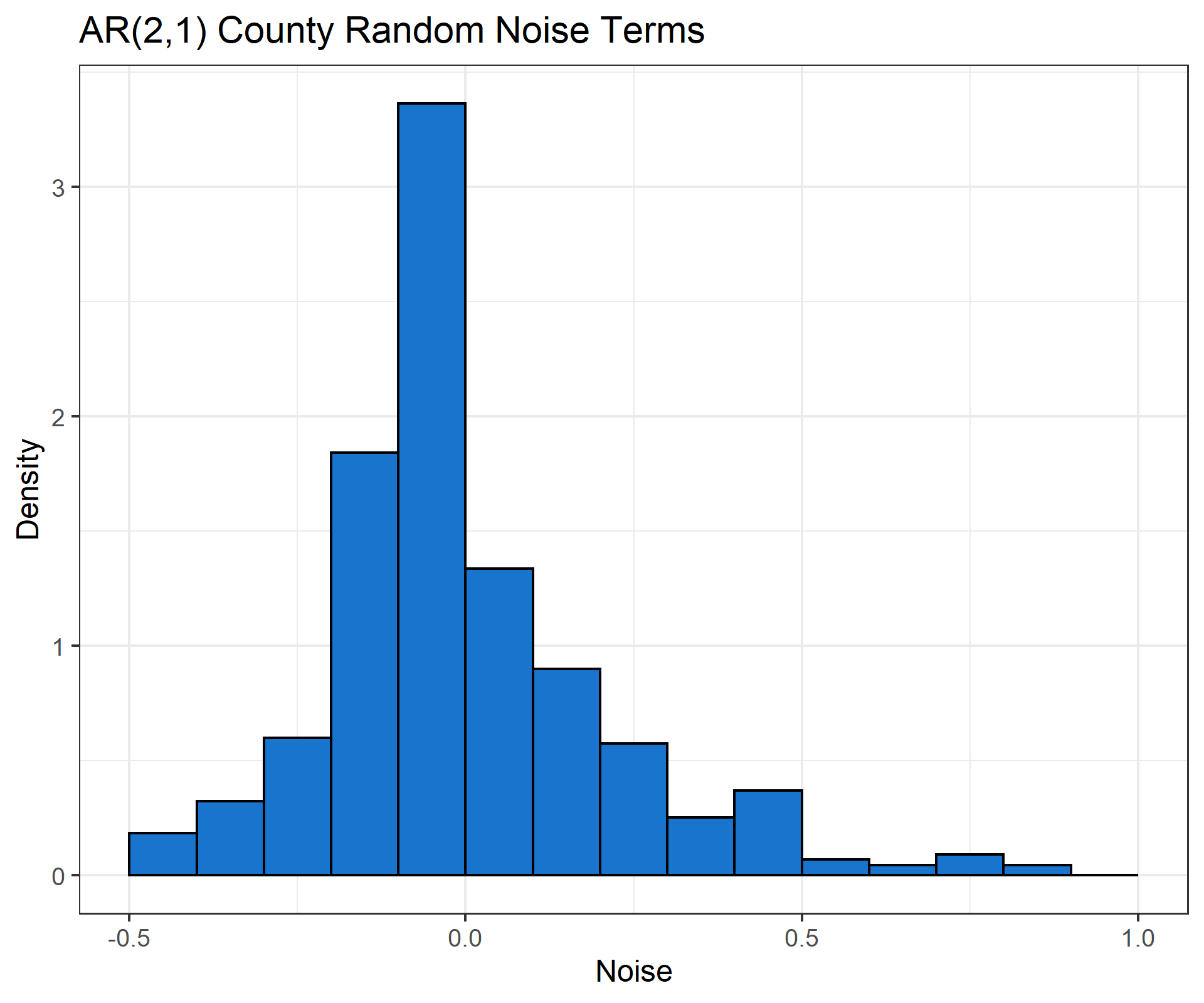

Supplement: S10 Fig — Density histogram of the reconstructed error terms for the spatial-temporal AR(2,1) model. (TIFF) [file pone.0275532.s013.tiff]

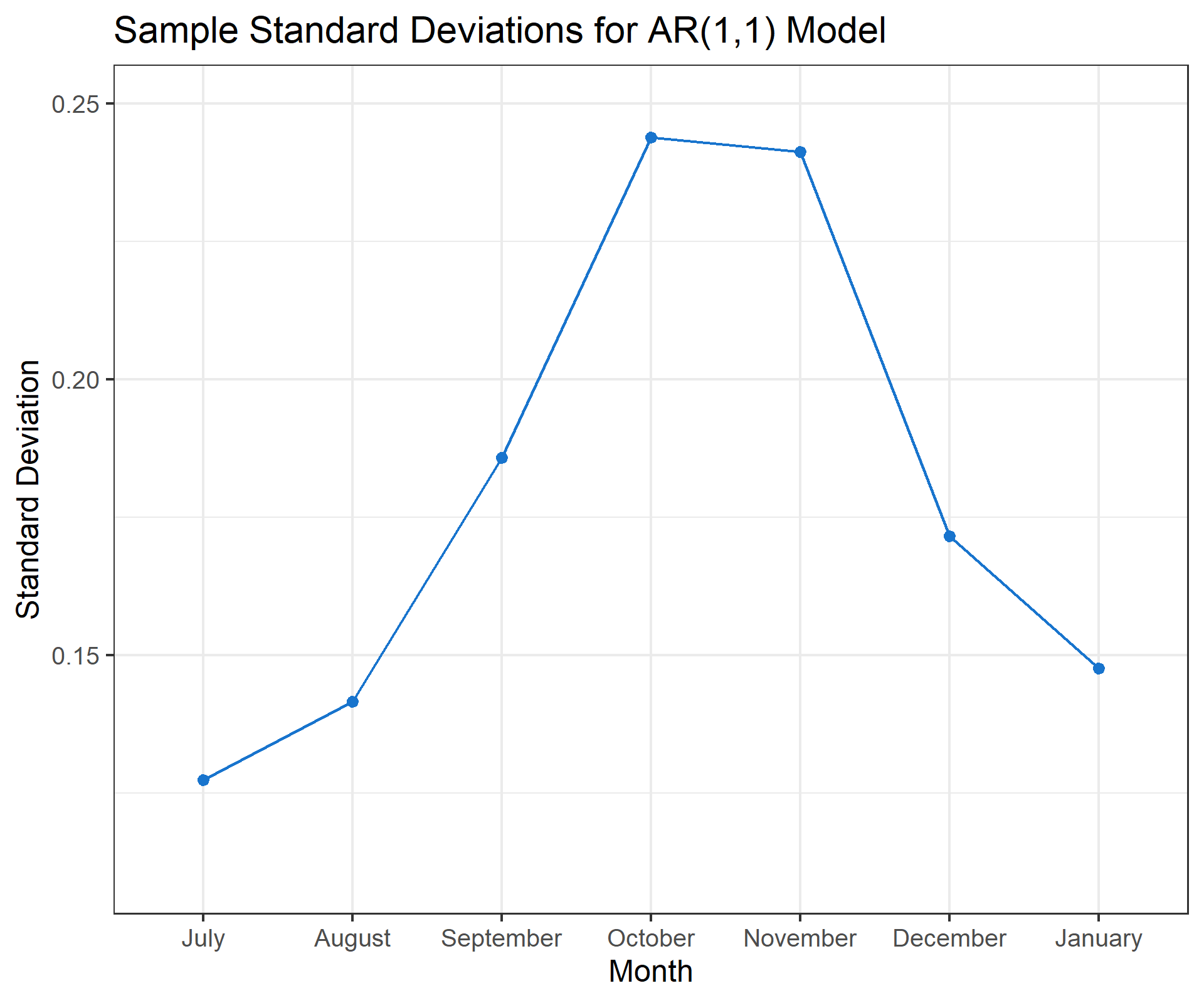

Supplement: S11 Fig — Monthly sample standard deviations of the reconstructed error terms for the spatial-temporal AR(1,1) model. (TIFF) [file pone.0275532.s014.tiff]

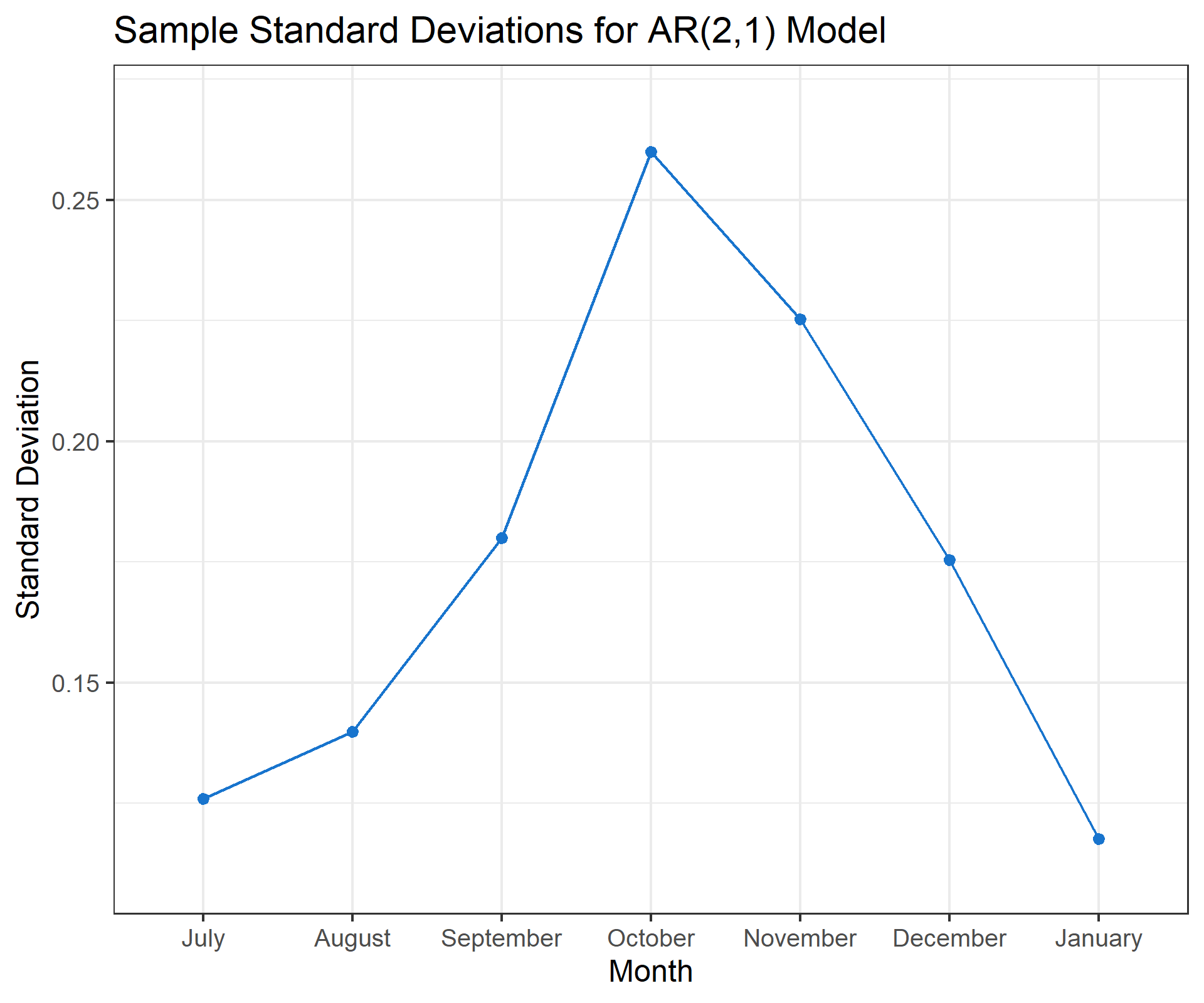

Supplement: S12 Fig — Monthly sample standard deviations of the reconstructed error terms for the spatial-temporal AR(2,1) model. (TIFF) [file pone.0275532.s015.tiff]
