# Supplementary material for: Testing network autocorrelation without replicates
Source: PLoS One. 2022 Nov 3;17(11):e0275532. doi: 10.1371/journal.pone.0275532 (PMC9632870; doi:10.1371/journal.pone.0275532)
Supplement: S1 Table — KS distances between simulated Q(K) and χK2 distributions for different values of n and K using the immuno dataset. (PDF) [file pone.0275532.s016.pdf]

S1 Table

Kolmogorov-Smirnov Distances						
	$K = 1$	$K = 2$	$K = 3$	$K = 4$	$K = 5$	$K = 6$
$n = 50$	0.0145	0.0344	0.0542	0.0708	0.0883	0.1076
$n = 100$	0.0057	0.0340	0.0610	0.0800	0.0960	0.1056
$n = 150$	0.0042	0.0204	0.0408	0.0604	0.0737	0.0835
$n = 200$	0.0045	0.0165	0.0342	0.0498	0.0585	0.0736
$n = 250$	0.0052	0.0174	0.0294	0.0442	0.0493	0.0613
$n = 300$	0.0064	0.0107	0.0205	0.0273	0.0419	0.0541
$n = 350$	0.0058	0.0084	0.0228	0.0334	0.0458	0.0545
$n = 400$	0.0063	0.0125	0.0182	0.0310	0.0417	0.0524
$n = 450$	0.0064	0.0138	0.0194	0.0280	0.0365	0.0449
$n = 500$	0.0044	0.0155	0.0180	0.0284	0.0376	0.0431
$n = 1000$	0.0075	0.0059	0.0077	0.0151	0.0165	0.0210

S1 Table. Kolmogorov-Smirnov distances for simulated test statistic distributions KS distances between simulated $Q(K)$ and χ^2_K distributions for different values of n and K using the immuno dataset.
