# Supplementary material for: Testing network autocorrelation without replicates
Source: PLoS One. 2022 Nov 3;17(11):e0275532. doi: 10.1371/journal.pone.0275532 (PMC9632870; doi:10.1371/journal.pone.0275532)
Supplement: S2 Table — Fitted spatial-temporal AR(p,1) models to logarithms of confirmed COVID-19 cases in New York counties. (PDF) [file pone.0275532.s017.pdf]

S2 Table

Fitted Spatial-Temporal Autoregressive Models						
	$\hat{\alpha}$	$\hat{\beta}_1$	$\hat{\beta}_2$	$\hat{\gamma}$	$\hat{\sigma}$	AIC
AR(1,1)	0.036	0.681	–	0.246	0.157	-311.251
AR(2,1)	0.018	0.455	0.309	0.208	0.154	-337.443

S2 Table. Fitted spatial-temporal autoregressive models. Fitted spatial-temporal AR(p,1) models to logarithms of confirmed COVID-19 cases in New York counties.
