# Supplementary material for: Testing network autocorrelation without replicates
Source: PLoS One. 2022 Nov 3;17(11):e0275532. doi: 10.1371/journal.pone.0275532 (PMC9632870; doi:10.1371/journal.pone.0275532)
Supplement: S3 Table — Obtained p-values for error terms of the spatial-temporal AR(p,1) model (n = 434). (PDF) [file pone.0275532.s018.pdf]

S3 Table

	AR(1,1)		AR(2,1)	
Spatial lags	$Q(K)$	p-value	$Q(K)$	p-value
$K = 1$	329.873	$p < 0.001$	265.814	$p < 0.001$
$K = 2$	547.247	$p < 0.001$	417.563	$p < 0.001$
$K = 3$	596.428	$p < 0.001$	440.301	$p < 0.001$
$K = 4$	599.586	$p < 0.001$	440.482	$p < 0.001$
$K = 5$	604.305	$p < 0.001$	450.905	$p < 0.001$
$K = 6$	608.769	$p < 0.001$	461.925	$p < 0.001$

S3 Table. Obtained p-values for spatial-temporal independence. Obtained p-values for error terms of the spatial-temporal AR(p,1) model ($n = 434$).
